# Supplementary material for: Massively parallel RNA device engineering in mammalian cells with RNA-Seq
Source: Nat Commun. 2019 Sep 23;10:4327. doi: 10.1038/s41467-019-12334-y (PMC6757056; doi:10.1038/s41467-019-12334-y)
Supplement: Supplementary file 1 — Supplementary Information [file 41467_2019_12334_MOESM1_ESM.pdf]

SUPPLEMENTARY INFORMATION

Massively Parallel RNA Device Engineering in Mammalian Cells with RNA-Seq

Xiang et al.

Supplementary Figures

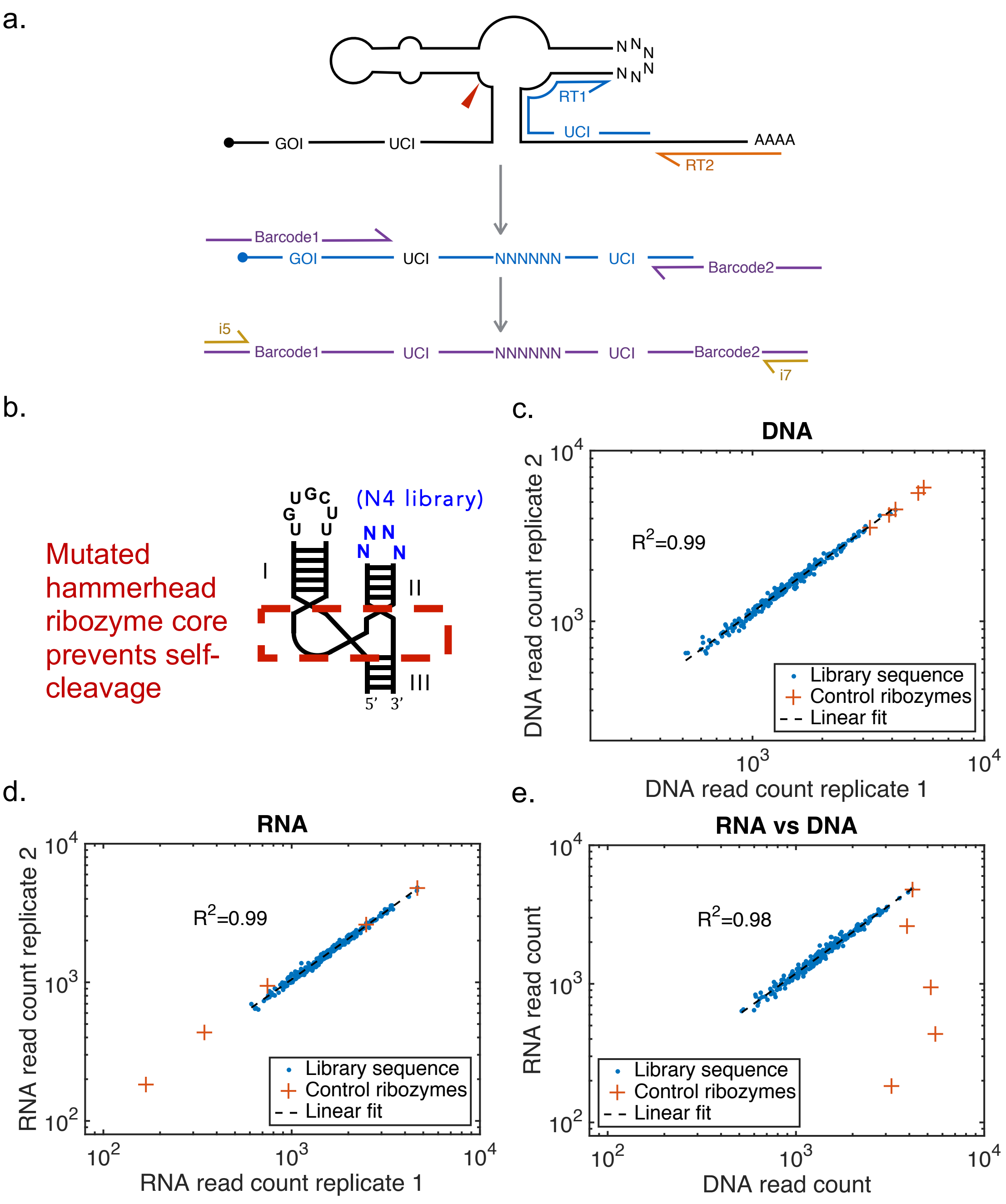

Supplementary Figure 1. RNA-Seq quantitatively measures raw RNA and DNA read counts for a control library of 256 unique sequences.
(a) A schematic illustrating the mechanism of reverse transcription and primer binding sites on the ribozyme switch sequence followed by library barcoding and Illumina adapter sequence PCR. UCI, unique coverage index.
(b) A schematic showing the non-cleaving ribozyme library, which consists of a mutated ribozyme core to prevent self-cleavage and a randomized loop II.

(c) DNA read count from replicate RNA-Seq experiments of the non-cleaving ribozyme library (R^2 = 0.99). Each dot represents a unique sequence with over 100 DNA read count. Pluses are spiked-in control ribozymes.
(d) RNA read count from replicate RNA-Seq experiments of the non-cleaving ribozyme library (R^2 = 0.99). Each dot represents a unique sequence with over 100 RNA read count.
(e) RNA read count plotted against DNA read count from an RNA-Seq experiment for the non-cleaving ribozyme library (R^2 = 0.98). Each dot is a unique sequence with over 100 RNA and DNA read count.
(c-e) All R^2 values are based on the raw read counts.

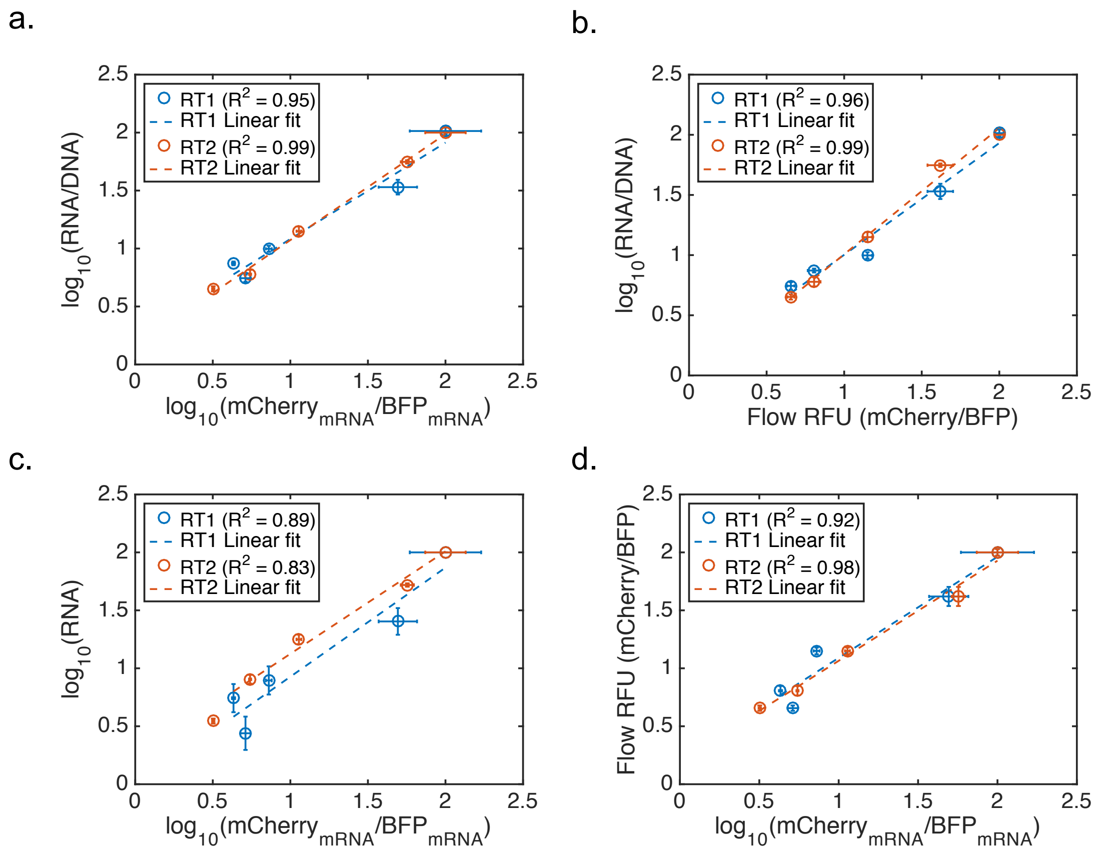

Supplementary Figure 2. RNA read counts from RNA-seq assays correlate with qPCR measurements of transcript levels and flow cytometry measurements of reporter fluorescence levels for control ribozymes.
(a) Normalized RNA read count from an RNA-Seq assay plotted against normalized mRNA levels from a qPCR assay. qPCR values are reported as mCherry transcript levels normalized to BFP transcript levels (a transfection control).
(b) Normalized RNA read count from an RNA-Seq assay plotted against relative fluorescence values of reporter protein expression, mCherry/BFP, from flow cytometry analysis of individual sequences.
(c) Unnormalized RNA read count from an RNA-Seq assay plotted against normalized mRNA levels from a qPCR assay.
(d) Relative fluorescence values of reporter protein expression, mCherry/BFP, from flow cytometry analysis of individual sequences plotted against normalized mRNA levels from a qPCR assay.
All values are $\log_{10}$ based on an arbitrary scale where sTRSVctl is set to 2. qPCR measurements are for three replicates, RNA-Seq measurements are for two or more replicates, and flow cytometry measurements are for four biological samples. Error bars indicate standard deviations of the replicates. Dashed lines are a result of a linear fit to the indicated data. RT1 and RT2 are primers used for reverse transcription, as indicated in Supplementary Figure 1a. All R^2 values are based on $\log_{10}$ transformed values.

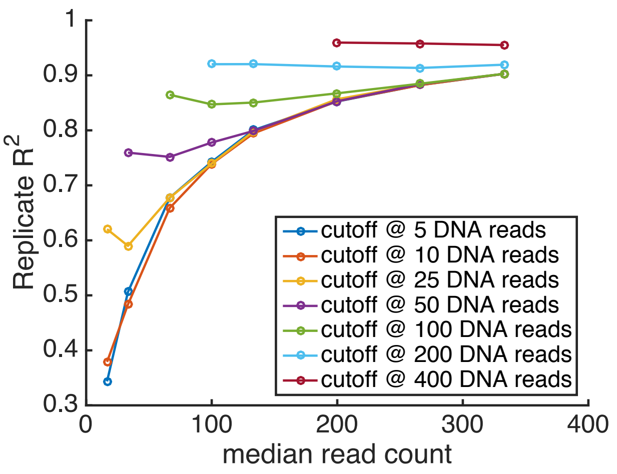

Supplementary Figure 3. Simulation of R^2 as a function of median library sequence read count and read count cutoff. The RNA-Seq data for the xanthine N5-6 library was used to generate the graph by random data sampling (without replacement) as performed in MATLAB. All R^2 values are based on $\log_{10}$ transformed normalized RNA values.

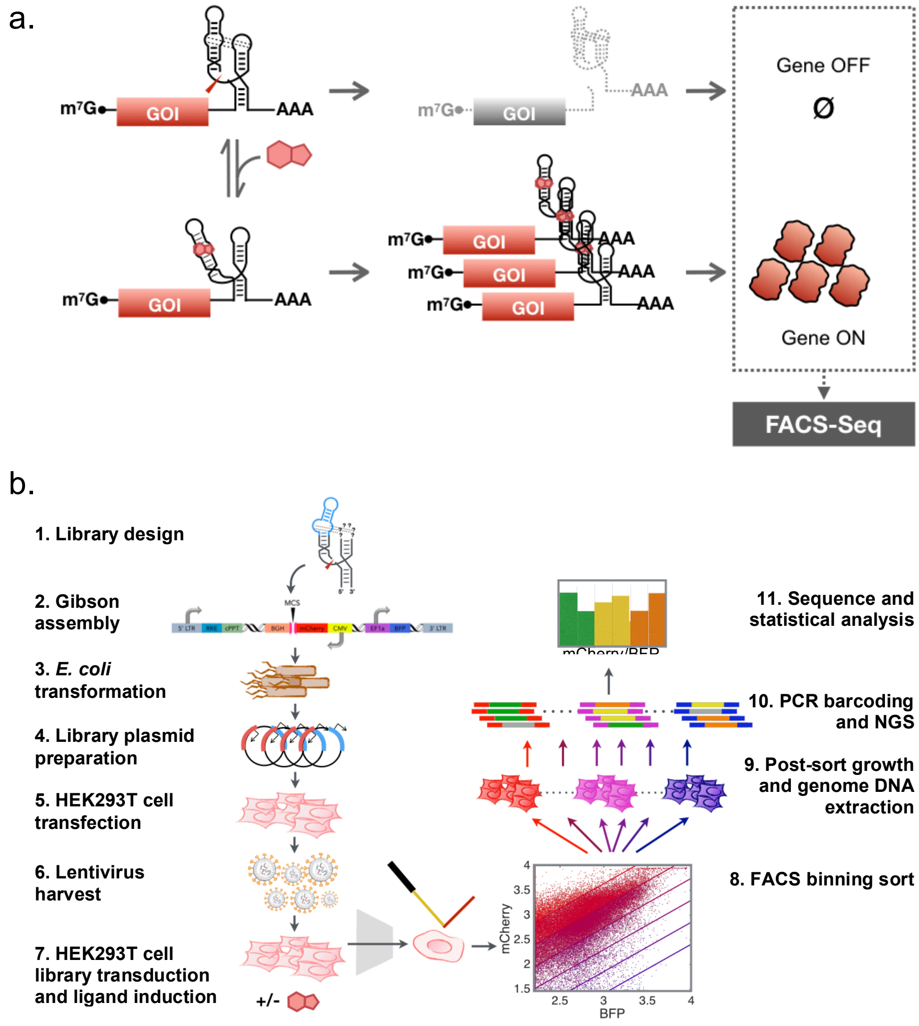

Supplementary Figure 4. Quantitative FACS-Seq for assaying protein expression levels as a result of ribozyme switch regulatory activity

(a) A schematic illustrating the mechanism by which ribozyme switches achieve conditional gene expression regulation, indicating that protein expression levels resulting from ribozyme switch activity can be assayed with FACS-Seq.

(b) FACS-seq workflow for measuring differential protein expression levels associated with a ribozyme switch library.

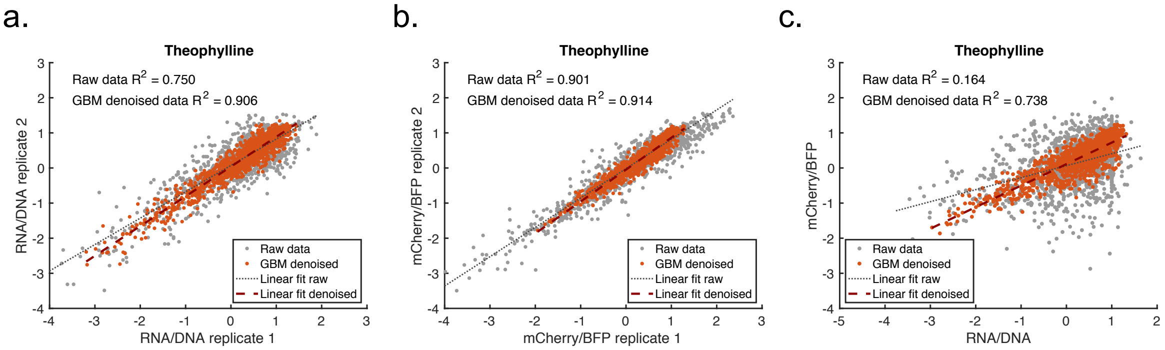

d.

GBM validation metric	RNA/DNA-seq 1	RNA/DNA-seq 2	FACS-seq 1	FACS-seq 2
R²	0.7877	0.7820	0.1967	0.2367
Mean squared error (MSE)	0.2094	0.1580	0.9327	0.6820
Mean absolute error (MAE)	0.3248	0.2928	0.6574	0.5686

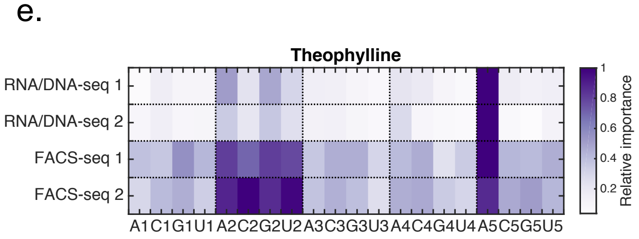

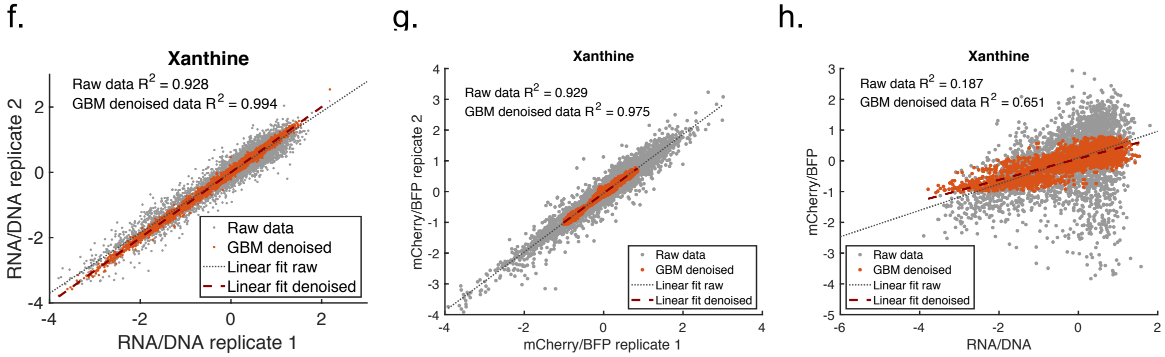

i.

GBM validation metric	RNA/DNA-seq 1	RNA/DNA-seq 2	FACS-seq 1	FACS-seq 2
R²	0.8534	0.8559	0.1445	0.1244
Mean squared error (MSE)	0.1299	0.1164	0.8079	0.7931
Mean absolute error (MAE)	0.2427	0.2384	0.6825	0.6713

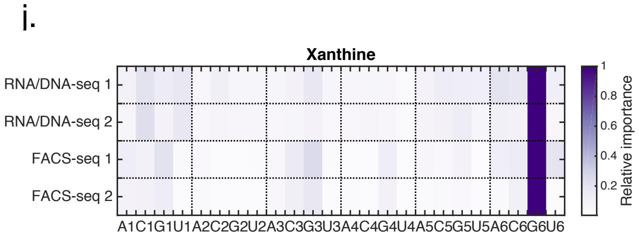

Supplementary Figure 5. RNA-Seq and FACS-Seq data denoised using Gradient Boosting Machine (GBM) ensemble regression implemented in H2O.ai.

(a) Normalized RNA read counts from replicate RNA-seq replicates experiments for 1,275 theophylline ribozyme switch library members with >20 read count coverage.

(b) Relative fluorescence values from replicate FACS-seq experiments for the 1,275 theophylline ribozyme switch library members with >20 read count coverage.

(c) Normalized RNA read count plotted against relative fluorescence values for the theophylline ribozyme switch library.

(d) GBM validation metrics showing performance of the converged model trained on the theophylline library data.

(e) Relative importance of loop II base position and identity in training the GBM on data from the RNA-seq and FACS-seq assays for the theophylline ribozyme switch library.

(f) Normalized RNA read counts from replicate RNA-seq replicates experiments for 5,069 xanthine ribozyme switch library members with >100 read count coverage.

(g) Relative fluorescence values from replicate FACS-seq experiments for the 5,069 xanthine ribozyme switch library members with >100 read count coverage.
(h) Normalized RNA read count plotted against relative fluorescence values for the xanthine ribozyme switch library.
(i) GBM validation metrics showing performance of the converged model trained on the xanthine library data.
(j) Relative importance of loop II base position and identity in training the GBM on data from the RNA-seq and FACS-seq assays for the xanthine ribozyme switch library.
(a-d, f-i) All R^2 values are based on $\log_{10}$ transformed values.

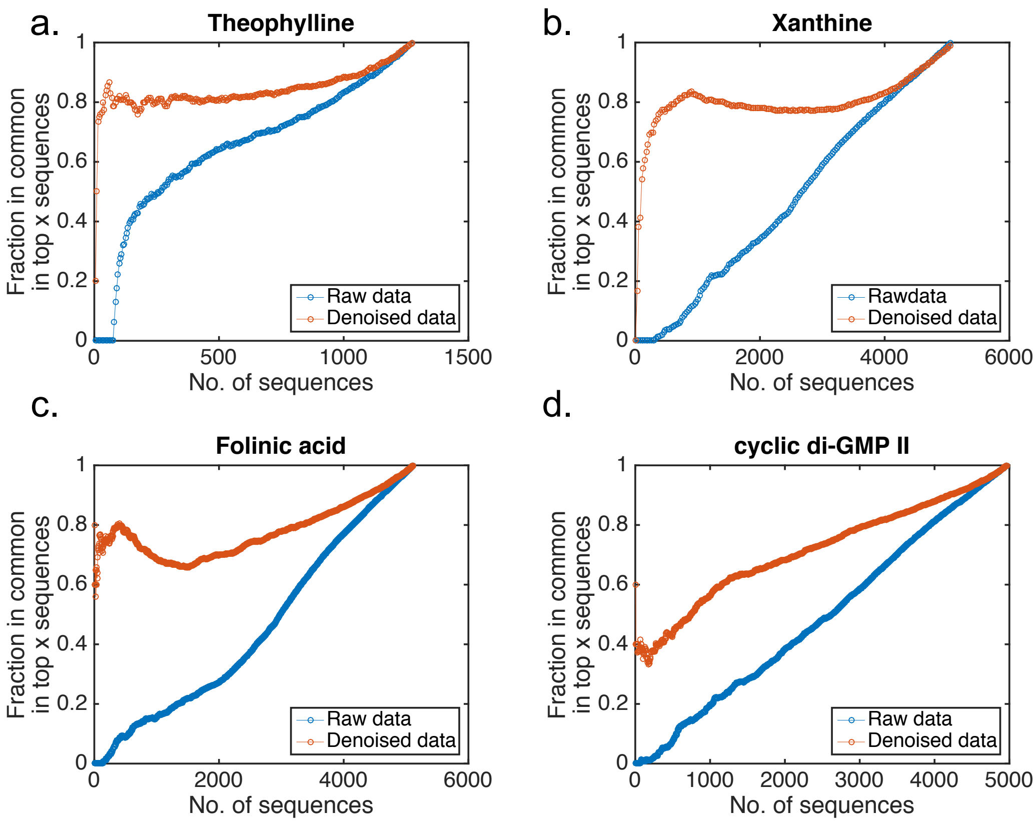

Supplementary Figure 6. The fraction of sequences shared between RNA-Seq and FACS-Seq results. Data is plotted as the top x sequences against the number of sequences. Ranking is by activation ratio. Results are shown for raw data (blue) and data denoised using AutoML (red), for (a) theophylline N4-5, (b) xanthine N5-6, (c) folinic acid N5-6, and (d) cyclic di-GMP-II N5-6 libraries in the mCherry vector pCS4076.

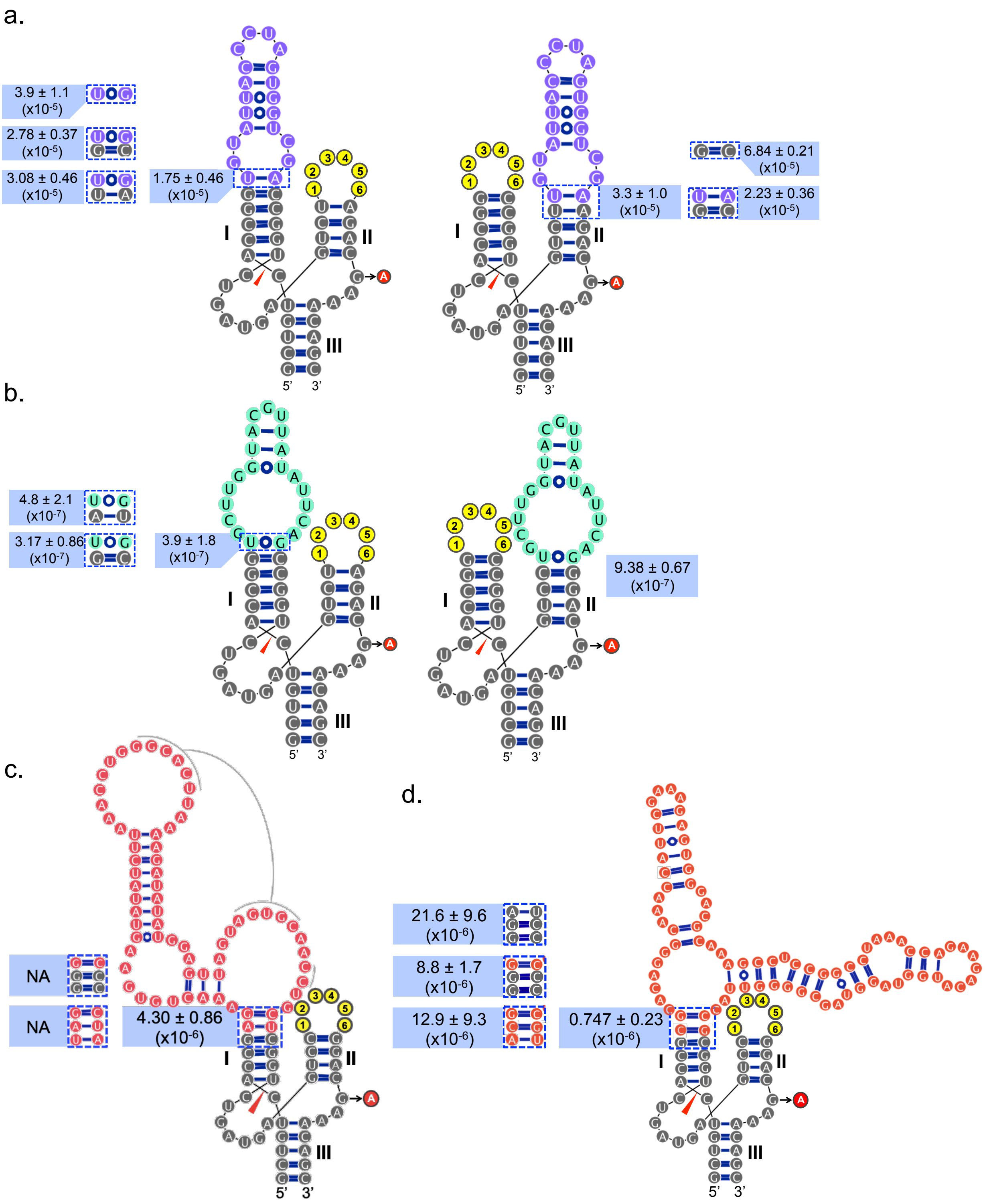

Supplementary Figure 7. Library designs integrating aptamer sequences (colored) with the sTRSV hammerhead ribozyme domain (gray). Equilibrium dissociation constants (K_D) of the aptamer integrated libraries as measured by surface plasmon resonance (SPR) binding affinity assays are indicated next to each design in shaded blue boxes. K_D values are in molar units (M), and the error reported is the standard error from the logistic regression fit. NA, not applicable or

no binding observed. Final library designs for each ligand group are the main figures in (a) left for xanthine, (b) left for folinic acid, (c) and (d) for cyclic di-GMP-II and cyclic di-GMP-I. Alternate tested designs are shown in blue dashed boxes, where the sequence differs from the final designs. Red circled nucleotide indicates a G to A mutation in the catalytic core to prevent ribozyme self-cleavage. Red narrow triangle indicates where the cleavage site would occur. Secondary structures are predicted from RNAstructure, with manual curation of tertiary interactions of the aptamer from crystal or NMR structures.

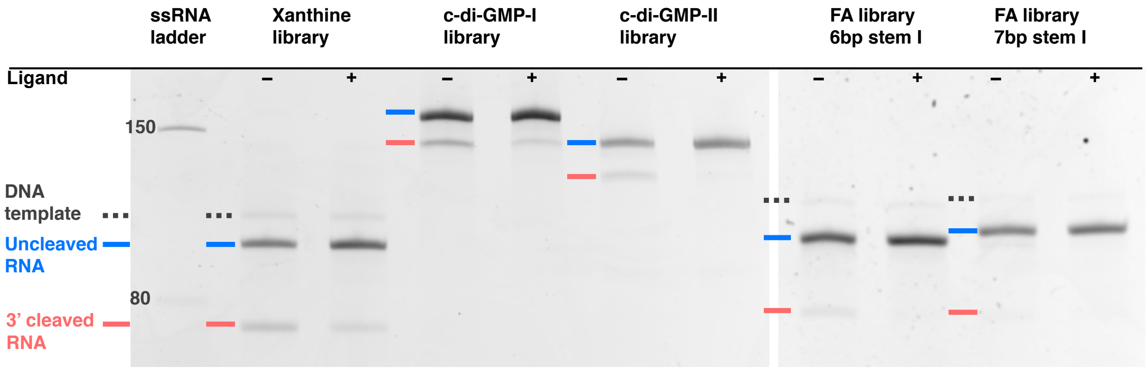

Library name	Xanthine		c-di-GMP-I		c-di-GMP-II		Folinic acid (6 bp stem I)		Folinic acid (7bp stem I)	
Ligand concentration (μM)	0	400	0	50	0	50	0	400	0	400
Fraction cleaved	31.5%	14.0%	23.3%	11.5%	21.7%	4.2%	10.6%	3.0%	5.6%	2.8%
Δ fraction uncleaved	1.26		1.15		1.22		1.08		1.03	
Δ fraction cleaved	2.25		2.03		5.23		3.49		2.00	

Supplementary Figure 8. Denaturing PAGE for xanthine, cyclic di-GMP, and folinic acid ribozyme switch libraries from *in vitro* transcribed RNA. Library RNA was synthesized in a co-transcriptional cleavage reaction and are indicated as a mixture of uncleaved (blue) and 3’ cleaved (red) RNAs on the gel. Each library is analyzed under two conditions, corresponding to -/+ ligand conditions in the transcription reaction and ligand concentrations are indicated in the table. Gels are stained with GelRed and darkness correlates with fluorescence intensity. The 80 and 150 nucleotide ssRNA ladder bands are indicated in the first lane.

a.

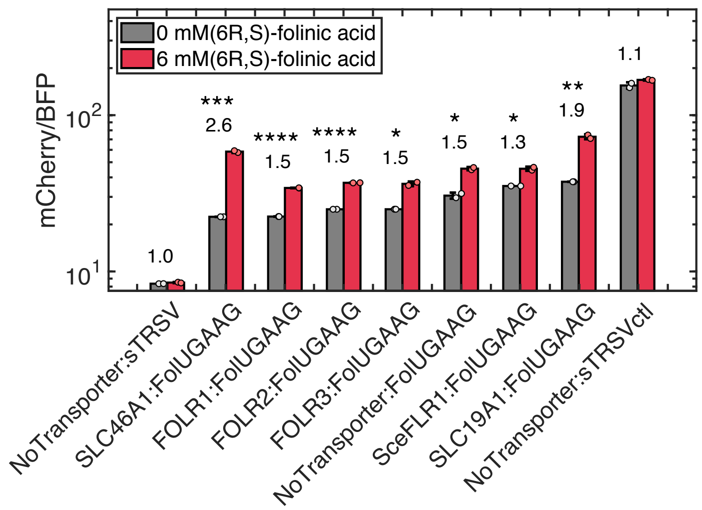

b.

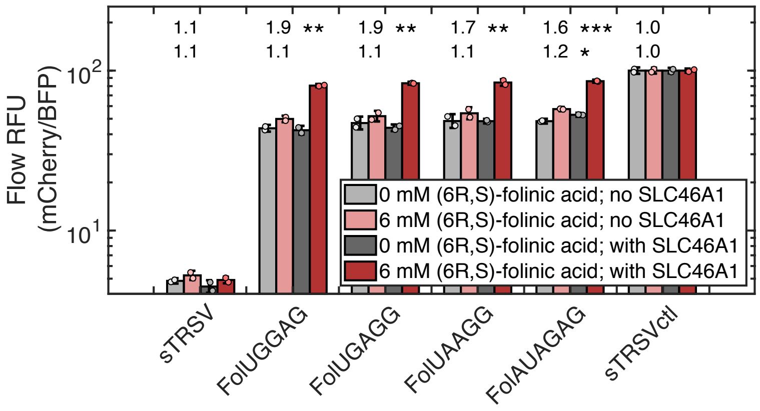

Supplementary Figure 9. Flow cytometry analysis to assay for folate transporter activity in inducing reporter gene expression from a folinic acid ribozyme switch.
(a) Relative fluorescence values (RFU) from flow cytometry analysis of HEK293T cells co-transfected with the indicated folate transporter and a control folinic acid ribozyme switch (FolUGAAG). Cells are incubated with 0 or 6 mM folinic acid for 48 hours. Labels above bars refer to activation ratio of the relative fluorescence values.
(b) Relative fluorescence values (RFU) from flow cytometry analysis of HEK293T cells that express the folate transporter, SLC46A1. Cells are transfected with the indicated folinic acid ribozyme switches and incubated with 0 or 6 mM folinic acid for 48 hours. Labels above bars refer to activation ratio; first row are results in a HEK293T cell line that stably expresses SLC46A1, the second row are results in a parental HEK293T cell line.
Error bars correspond to standard deviation of two biological replicates. Asterisks indicate the Benjamini-Hochberg positive false discovery rate of switching significance, using p-values from unpaired, one-tailed t-test, * 5%, ** 0.5%, *** 0.05%, **** 0.005%. sTRSV is the wild-type hammerhead ribozyme; sTRSVctl is a non-cleaving mutant of sTRSV. Filled circles are individual replicate data points.

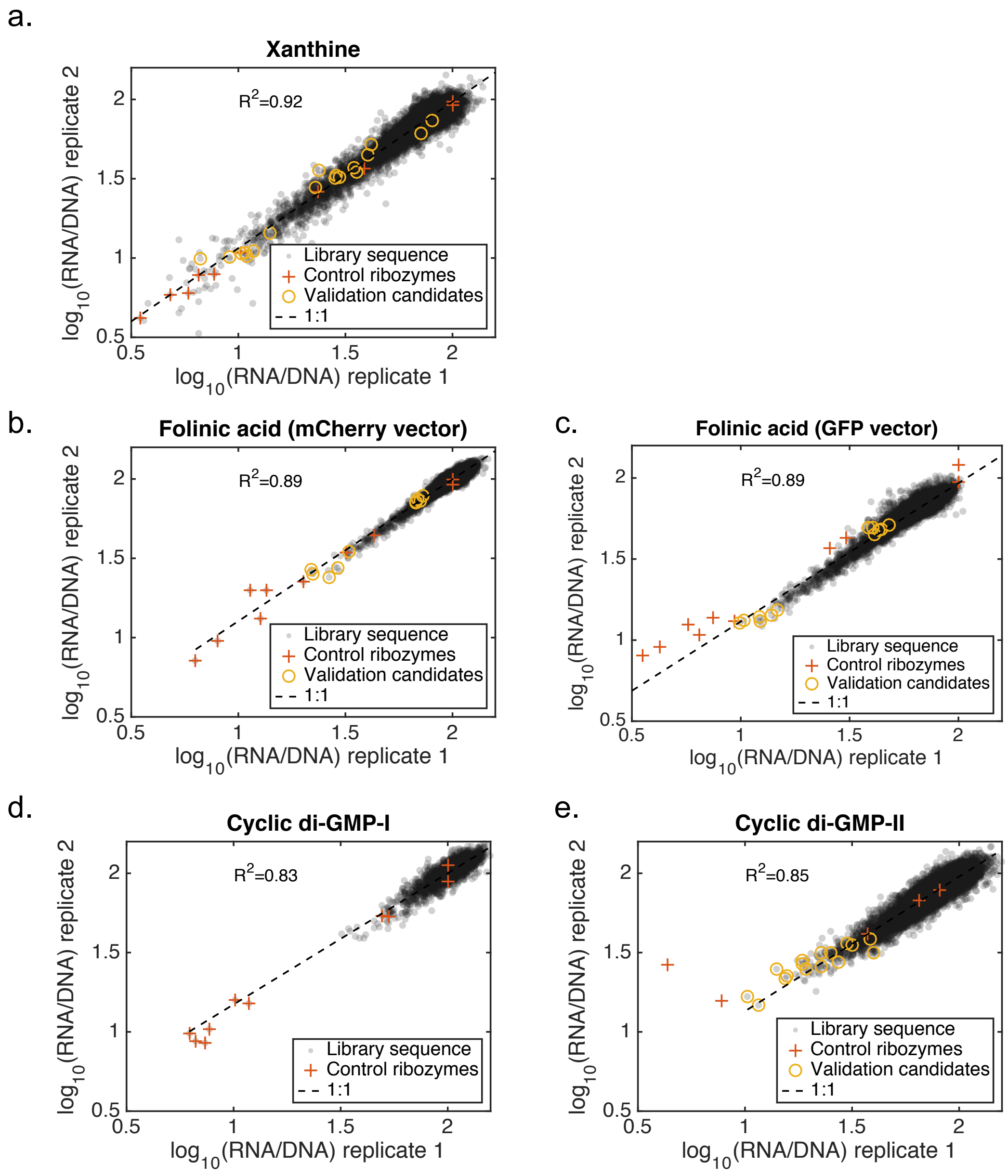

Supplementary Figure 10. Normalized RNA read counts from replicate RNA-Seq experiments. (a) 5,083 unique sequences characterized for the xanthine N5-6 library ($R^2 = 0.92$), (b) 5,124 unique sequences characterized for the folinic acid N5-6 library in the mCherry vector ($R^2 = 0.89$), (c) 5,171 unique sequences characterized for the folinic acid N5-6 library in the GFP vector ($R^2 = 0.89$), (d) 1,006 unique sequences characterized for the cyclic-di-GMP-I N5 library ($R^2 = 0.83$), (e) 4,961 unique sequences characterized for the cyclic-di-GMP-II N5-6 ($R^2 = 0.85$). Each dot represents a unique sequence with over 100 sequencing read counts in an experiment. Pluses are spiked-in control ribozymes; circles indicate sequences selected for validation. All R^2 values are based on $\log_{10}$ transformed normalized RNA values.

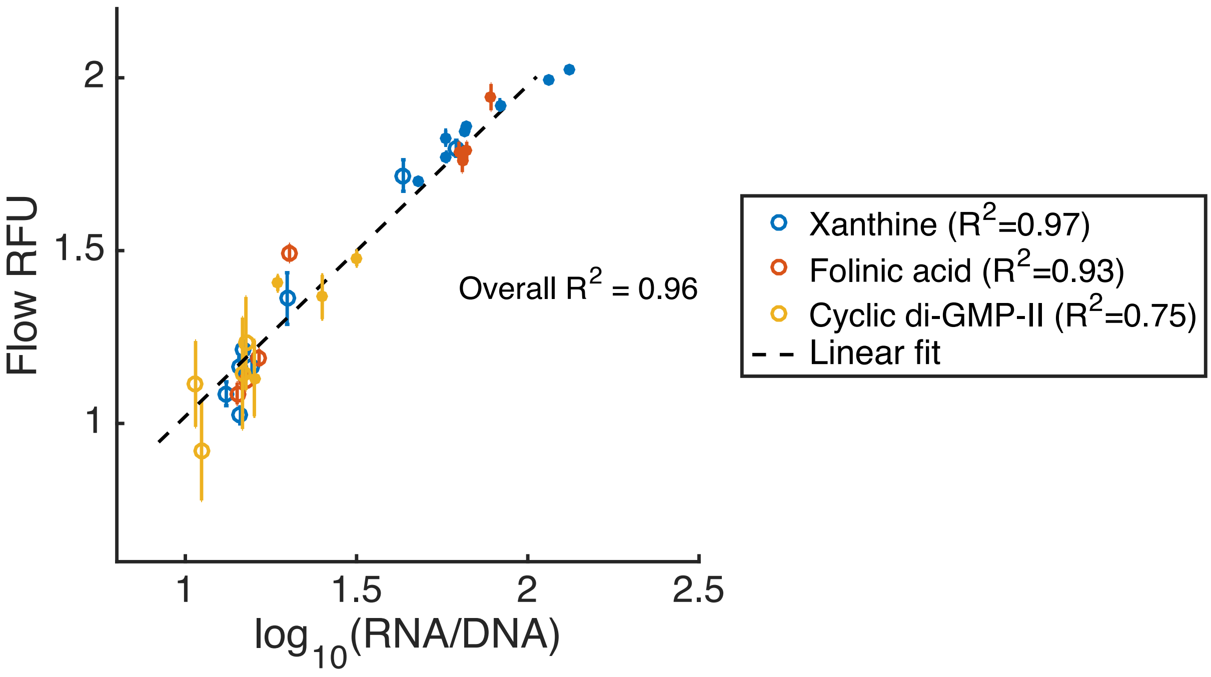

Supplementary Figure 11. Relative fluorescence values from flow cytometry analysis of individual sequences plotted against normalized RNA read counts from RNA-Seq assays. Dashed line represents a linear fit to the data ($R^2 = 0.96$). Open circles, without ligand; filled circles, with ligand. Error bars are standard deviations of two biological replicates. All R^2 values are based on log_{10} transformed normalized RNA values.

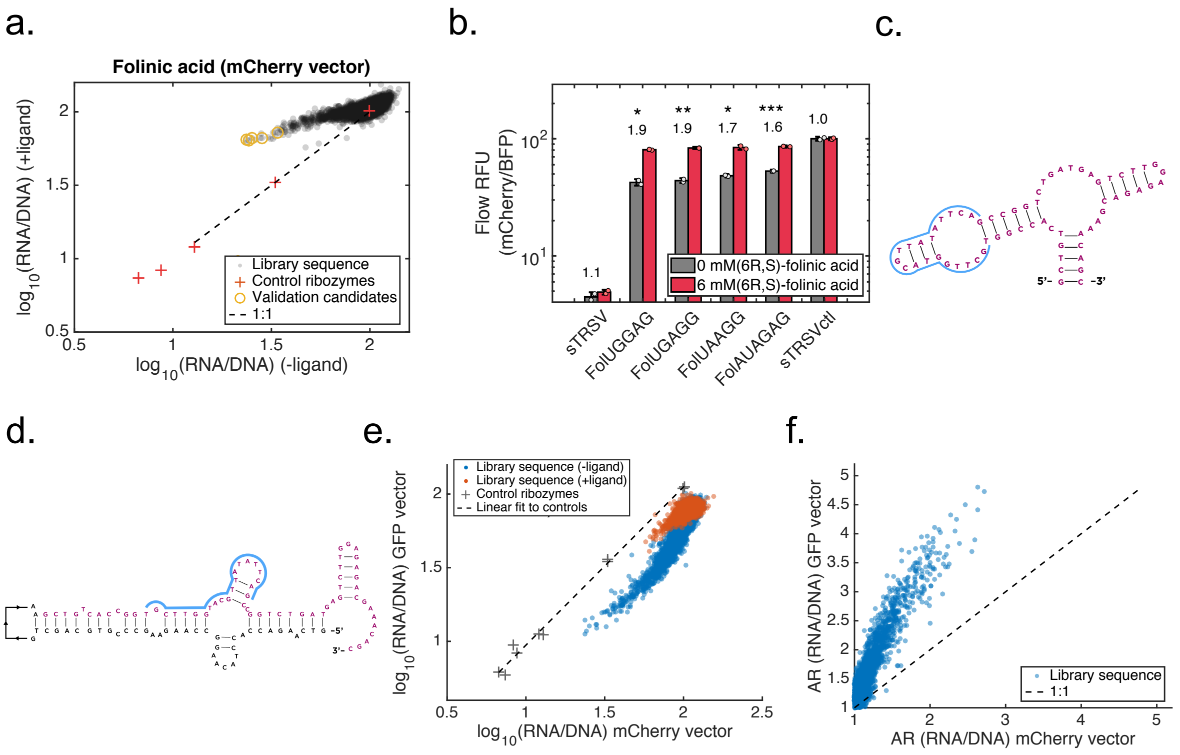

Supplementary Figure 12. Context dependent folding of the folinic acid ribozyme switches. (a) Normalized RNA read counts from an RNA-Seq assay performed on a folinic ribozyme switch library in the absence and presence of ligand in HEK293T cells overexpressing the SLC46A1 folate transporter. Each dot represents a unique sequence with over 100 sequencing read counts in an experiment. Pluses are spiked-in control ribozymes; circles are sequences selected for validation; -/+ ligand conditions are 0/6 mM (6R,S)-folinic acid. Dashed line is a 1:1 ratio of the values of the two axes.
(b) Flow cytometry analysis of individual members in the folinic acid ribozyme switch library. Activation ratio of mCherry/BFP is indicated above each set of bars. sTRSV is the wild-type hammerhead ribozyme; sTRSVctl is a non-cleaving mutant of sTRSV; asterisks indicate the Benjamini-Hochberg positive false discovery rate of switching significance, using p-values from unpaired, one-tailed t-test, * 5%, ** 0.5%, *** 0.05%, **** 0.005%; error bars indicate standard deviation of two biological replicates. Filled circles are individual replicate data points.
(c) Predicted secondary structure of the properly folded folinic acid ribozyme switch FolUGGAG as predicted by RNAstructure. Blue line indicates the folinic acid aptamer.
(d) Predicted secondary structure of the folinic acid switch FolUGGAG in the context of the mCherry transcript. Blue line indicates the folinic acid aptamer. Red, sequence associated with the sTRSV ribozyme; black, sequence associated with the mCherry coding sequence.
(e) Normalized RNA read counts from an RNA-Seq assay performed on the folinic acid ribozyme switch library in the context of the eGFP reporter transcript plotted against the normalized RNA read counts in the context of the mCherry reporter transcript. Each dot represents a unique sequence; dashed line is a linear fit to the control ribozymes.
(f) Activation ratio of normalized RNA read counts for the folinic acid ribozyme switch library in the context of the eGFP reporter transcript plotted against the activation ratio in the context of the mCherry reporter transcript. Dashed line is a 1:1 ratio of the values of the two axes.

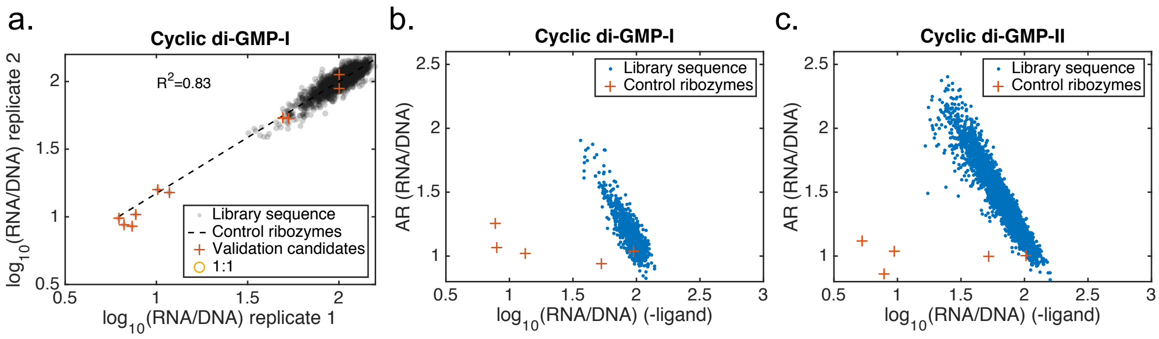

Supplementary Figure 13. RNA-Seq analysis of cyclic di-GMP libraries.
(a) Normalized RNA read counts from an RNA-seq assay performed on a cyclic di-GMP-I ribozyme switch library in the absence and presence of ligand. Each dot represents a unique sequence with over 100 sequencing read counts (1,011 sequences). Pluses are spiked-in control ribozymes; -/+ ligand conditions are 0/1 mM cyclic di-GMP.
(b) Activation ratio as a function of normalized RNA read counts in the absence of ligand for the cyclic di-GMP-I ribozyme switch library.
(c) Activation ratio as a function of normalized RNA read counts in the absence of ligand for the cyclic di-GMP-II ribozyme switch library. Each dot is a unique sequence with over 100 sequencing read counts (4,966 sequences).

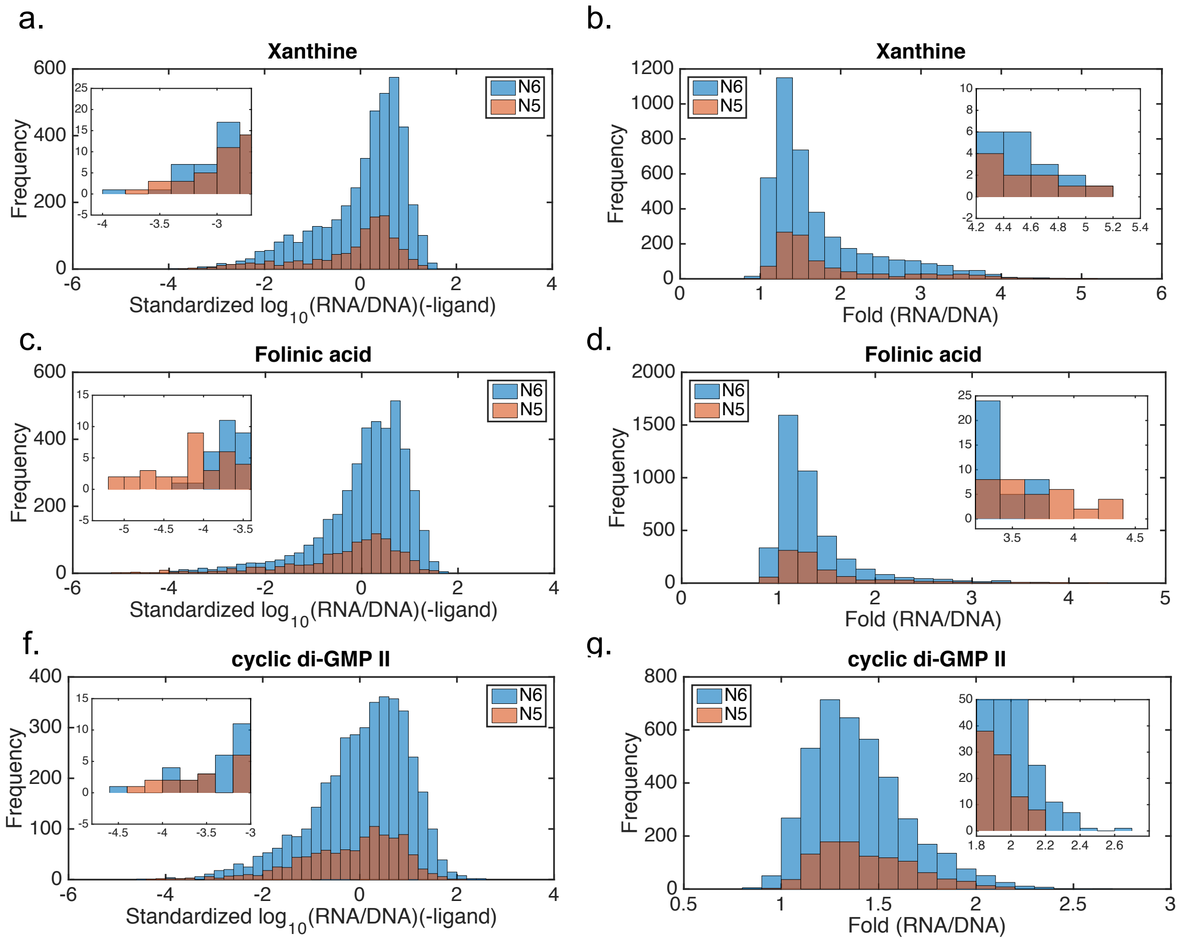

Supplementary Figure 14. Distribution of standardized, normalized RNA read counts and activation ratios of normalized RNA read counts.

(a) Histogram of standardized, normalized RNA read counts from an RNA-Seq assay for N5 and N6 loop II sequences of the xanthine ribozyme switch library. Inset zooms into the sequences with the lowest normalized RNA read counts.

(b) Activation ratios of normalized RNA read counts from an RNA-Seq assay for N5 and N6 loop II sequences of the xanthine ribozyme switch library. Inset zooms into the sequences with the highest activation ratios.

(c) Histogram of standardized, normalized RNA read counts from an RNA-Seq assay for N5 and N6 loop II sequences of the folinic acid ribozyme switch library. Inset zooms into the sequences with the lowest normalized RNA read counts.

(d) Activation ratios of normalized RNA read counts from an RNA-Seq assay for N5 and N6 loop II sequences of the folinic acid ribozyme switch library. Inset zooms into the sequences with the highest activation ratios.

(e) Histogram of standardized, normalized RNA read counts from an RNA-Seq assay for N5 and N6 loop II sequences of the cyclic di-GMP-II ribozyme switch library. Inset zooms into the sequences with the lowest normalized RNA read counts.

(f) Activation ratios of normalized RNA read counts from an RNA-Seq assay for N5 and N6 loop II sequences of the cyclic di-GMP-II ribozyme switch library. Inset zooms into the sequences with the highest activation ratios.

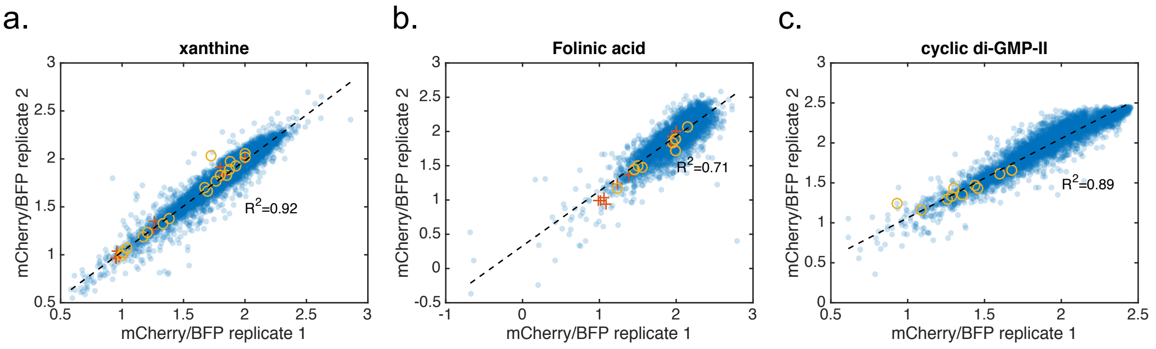

Supplementary Figure 15. Replicate FACS-seq experiments of new ribozyme switch libraries.

(a) Relative fluorescence values from replicate FACS-Seq experiments ($R^2 = 0.92$) performed on a xanthine ribozyme switch library. Each dot represents a unique sequence with over 100 sequencing read counts in an experiment (2,550 sequences). Pluses are spiked-in control ribozymes; circles are sequences selected from RNA-Seq for validation.

(b) Relative fluorescence values from replicate FACS-Seq experiments ($R^2 = 0.71$) performed on a folinic acid ribozyme switch library. Each dot represents a unique sequence with over 100 sequencing read counts in an experiment (1,784 sequences).

(c) Relative fluorescence values from replicate FACS-Seq experiments ($R^2 = 0.89$) performed on a cyclic di-GMP-II ribozyme switch library. Each dot represents a unique sequence with over 100 sequencing read counts in an experiment (2,293 sequences).

All R^2 values are based on $\log_{10}$ transformed relative fluorescence values.

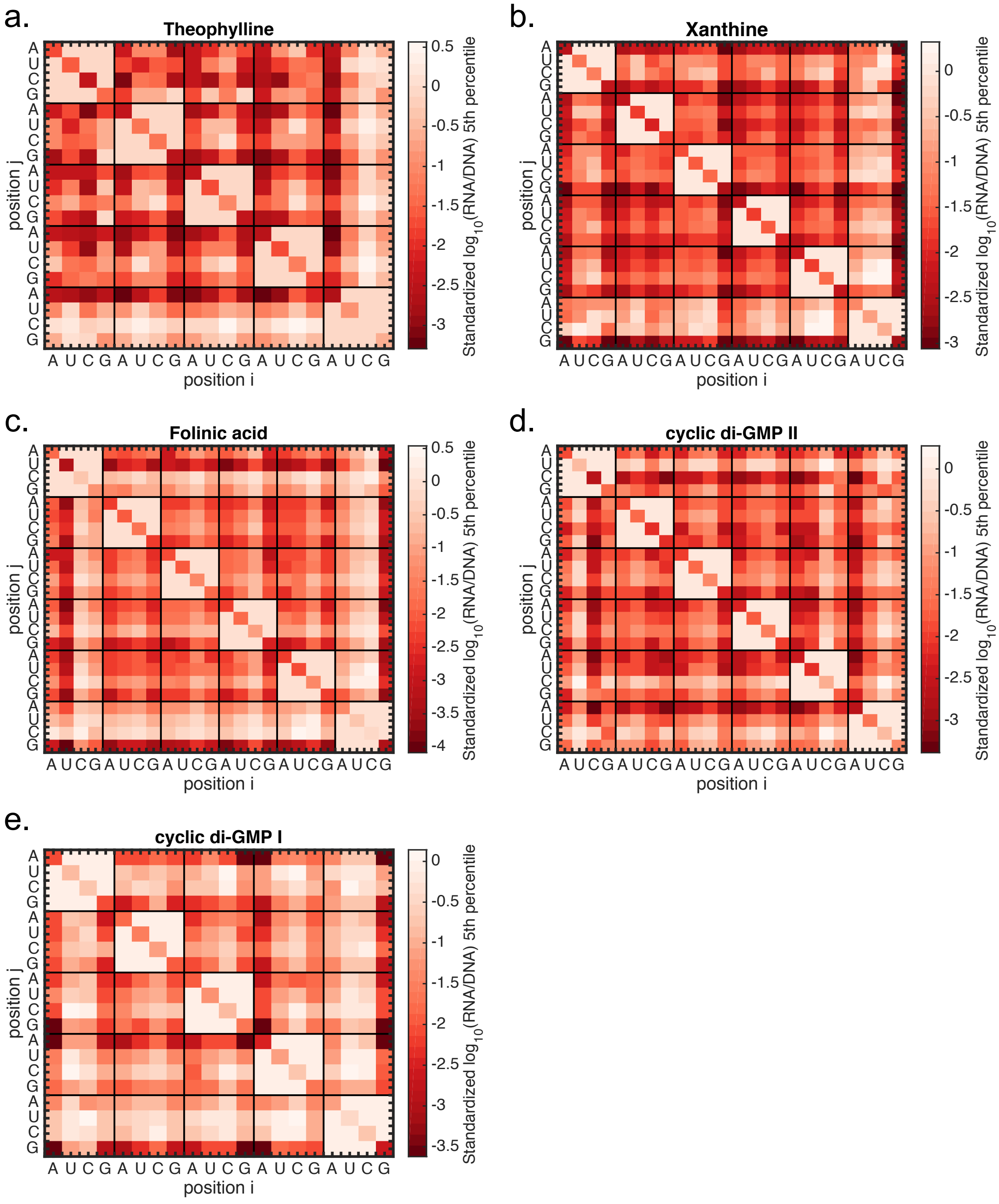

Supplementary Figure 16. Pairwise contribution to the 5th percentile standardized log$_{10}$(RNA/DNA) of the library sequence for each of A, U, C, G nucleotides at two positions, *i* and *j*, within loop II. Results are shown for (a) theophylline N4-5, (b) xanthine N5-6, (c) folinic acid N5-6, (d) cyclic di-GMP-II N5-6, and (e) cyclic di-GMP-I N5 ribozyme switch libraries using data from RNA-seq.

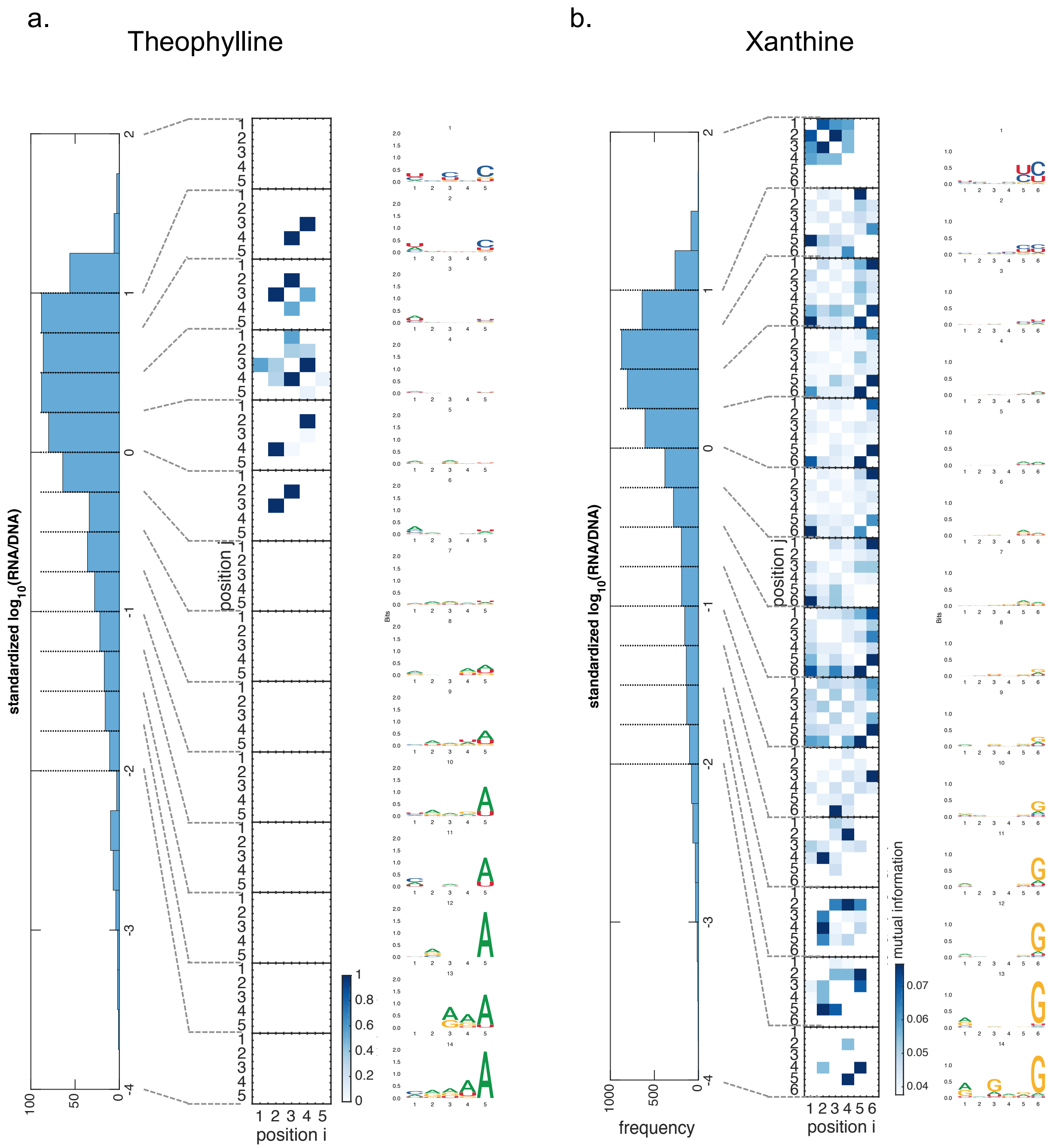
a.
Theophylline
b.
Xanthine
standardized log10(RNA/DNA)
position j
position i
frequency
mutual information
Bits

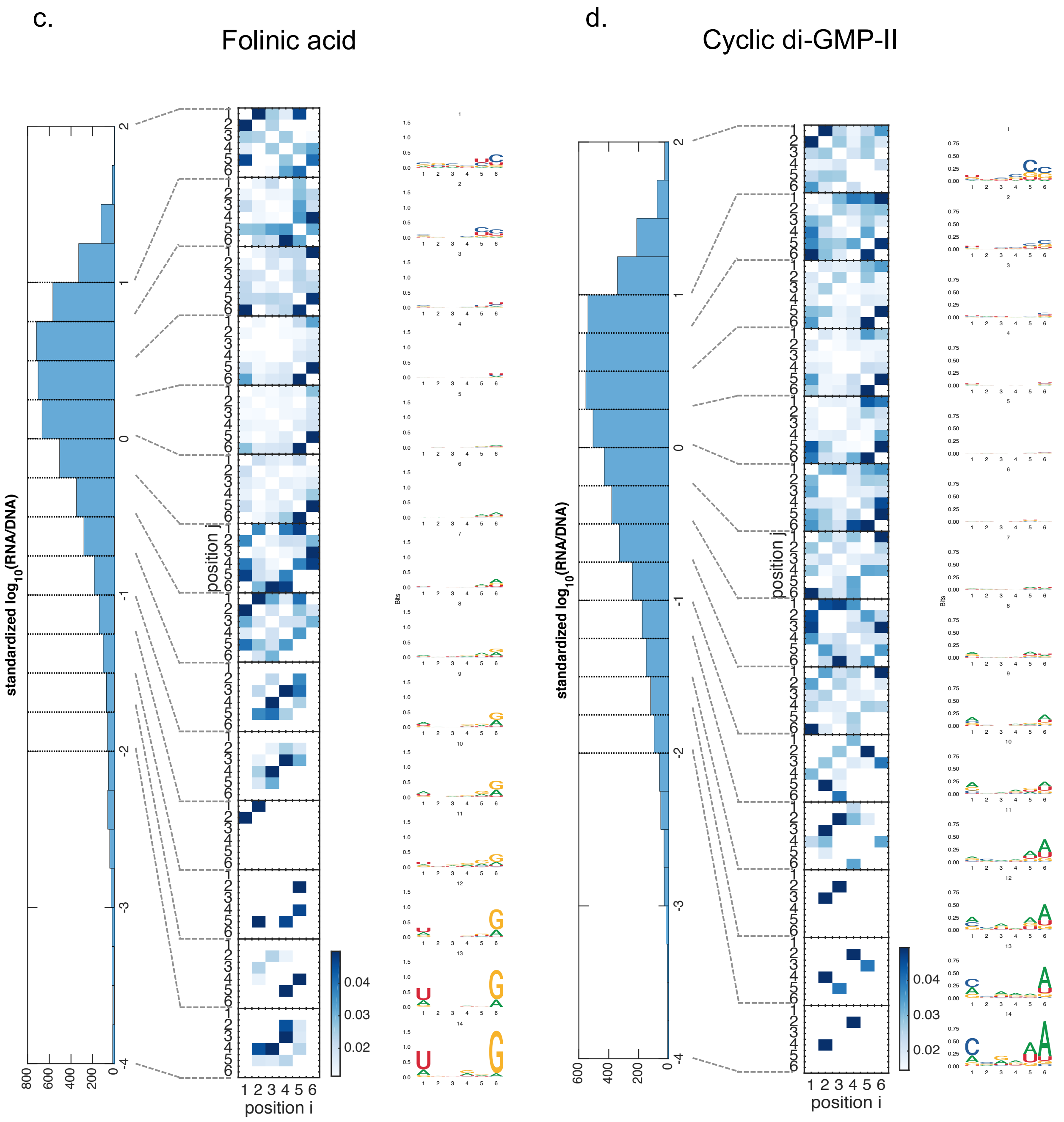

c.
Folinic acid
d.
Cyclic di-GMP-II
standardized log10(RNA/DNA)
position j
position i
0.04
0.03
0.02
Bits

e.

Cyclic di-GMP-I

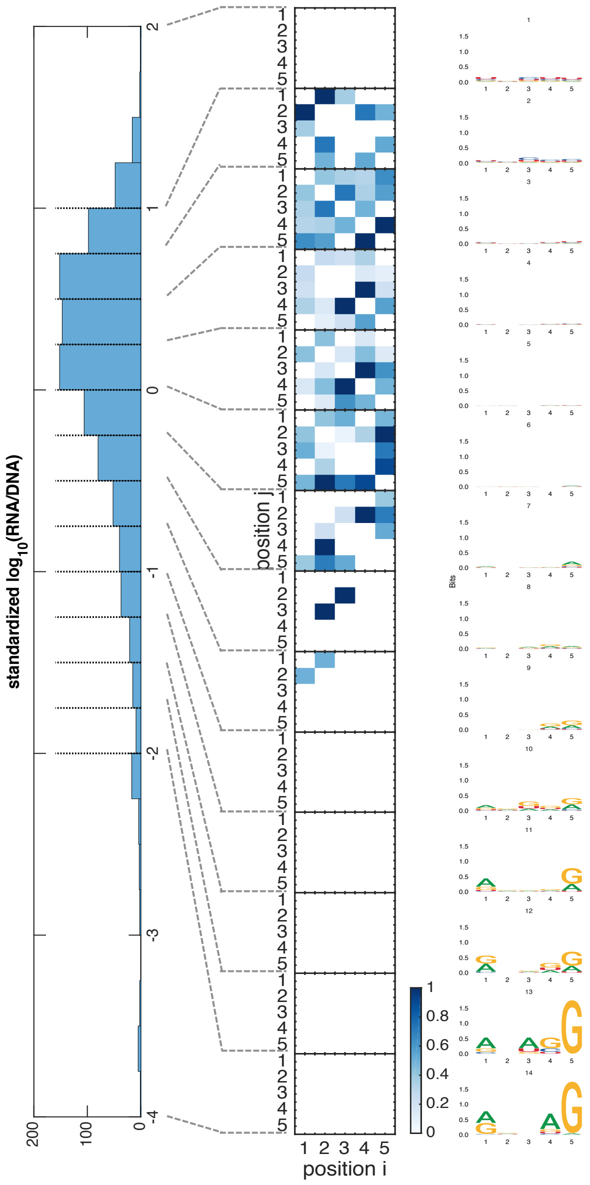

Supplementary Figure 17. Sequence and structural motifs of sequences in activity bins from histograms of standardized, normalized RNA read counts obtained from RNA-seq. Each ribozyme switch library is divided into 14 activity bins according to different levels of standardized $\log_{10}$(RNA/DNA). For sequences in each bin, mutual information analysis is indicated for a pair of nucleotides in loop II in the heat map, and the sequence logo of the sequences in the same bin is shown to the right. The results of this analysis are shown for the (a) theophylline N4-5, (b) xanthine N5-6, (c) folinic acid N5-6, (d) cyclic di-GMP-II N5-6, and (e) cyclic di-GMP-I N5 ribozyme switch libraries.

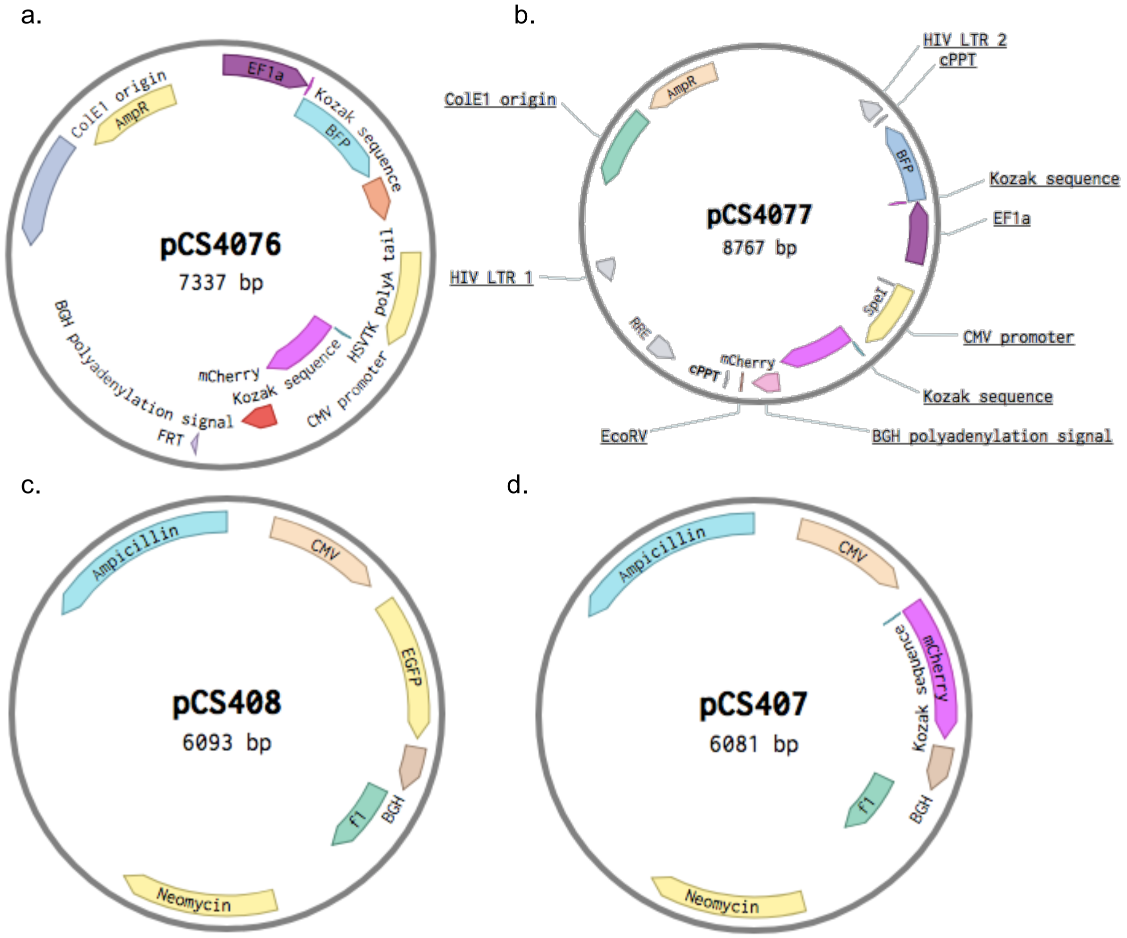

Supplementary Figure 18. Key plasmid maps for experiments described in this work. (a) pCS4076 is used for transient transfection and RNA-seq experiments. (b) pCS4077 is used for lentiviral library transduction experiments. (c) pCS408 is used for folinic acid ribozyme switch RNA-seq library experiments and transient co-transfection experiments with pCS407. (d) pCS407 is used as a transfection control with pCS408 in co-transfection experiments. Ribozyme switches and control ribozymes are inserted into pCS4076, pCS4077, and pCS408 right after the stop codon for the fluorescent reporter (mCherry, mCherry, and EGFP, respectively), and before the start of the polyA sequence.

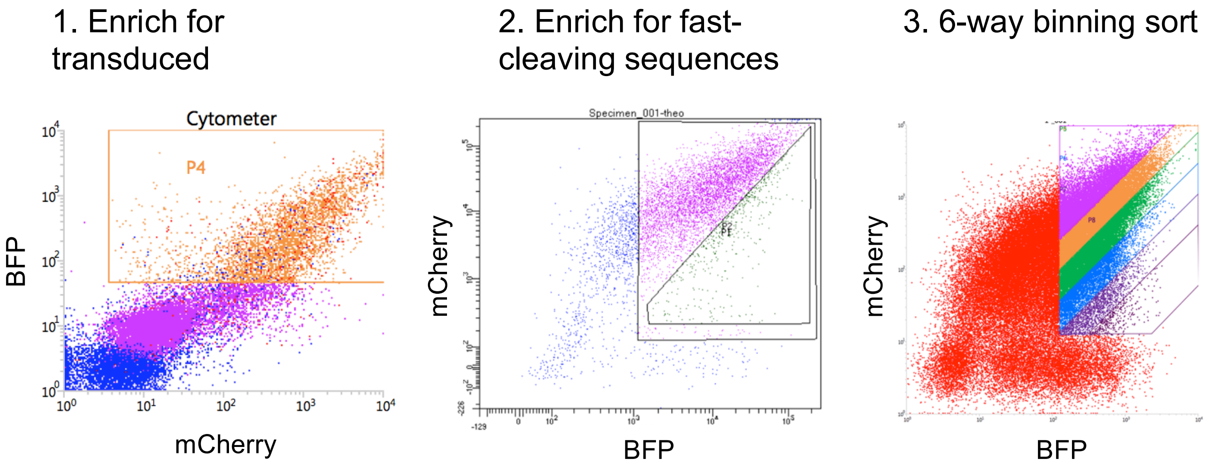

Supplementary Figure 19. Example sort plots showing the three stages of enrichment for the ribozyme switch libraries. 1) ~10-20% of the transduced library is sorted (orange gate); 2) ~10% of the fast-cleaving sequences are enriched (green gate); and 3) the final 6-way binning sort (non-red gates) performed on the BD Influx FACS sorter.

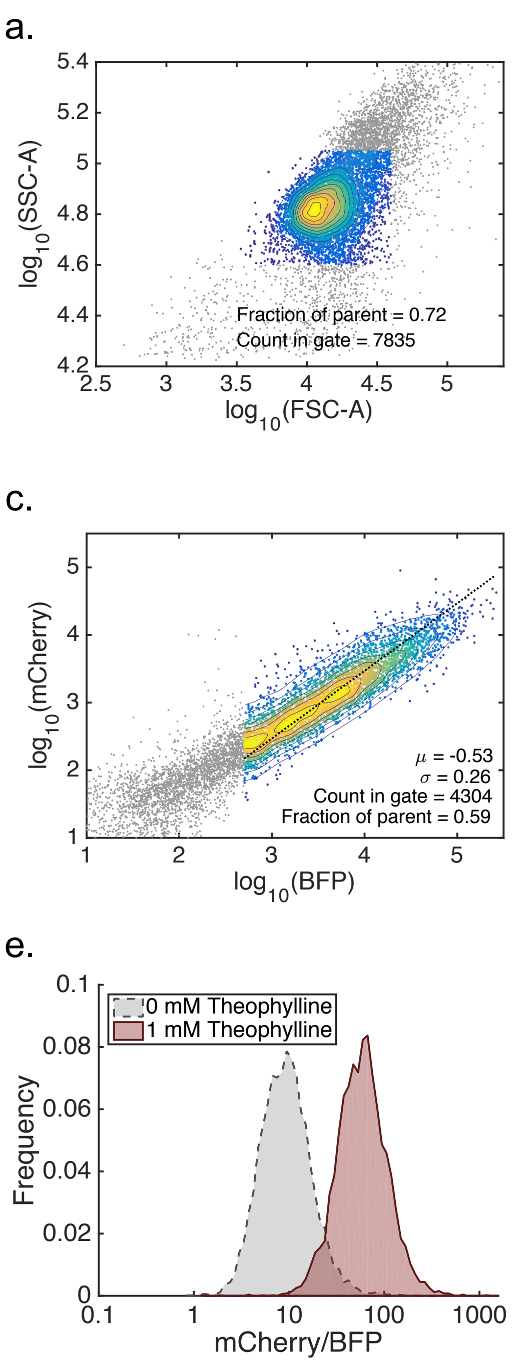

Supplementary Figure 20. Representative density-contour plots of flow cytometry data for a theophylline ribozyme switch (TheoAAAAA).

(a) Side scatter area (SSC-A) plotted against forward scatter area (FSC-A) for the starting population of individual flow data events (grey dot); events are gated for viable cells, defined as $3.5<\log_{10}$(FSC-A)<4.6, and $4.6< \log_{10}$(SSC-A)<5.05, and are represented in the density-contour region.

(b) $\log_{10}$(FSC-H) plotted against $\log_{10}$(FSC-A) for the viable cells from (a) (grey dot); cells are gated for singlets, defined as $\log_{10}$(FSC-H)$<0.65*\log_{10}$(FSC-A), and are represented in the density-contour region.

(c) For TheoAAAAA in the 0 mM theophylline condition, fluorescence values $\log_{10}$(mCherry) are plotted against $\log_{10}$(BFP) for singlet cells (grey dot); cells are gated for transfected, defined as $\log_{10}$(BFP)>2.7, and are represented in the density-contour region. $\log_{10}$(mCherry/BFP) is fit to a Gaussian distribution, with mean μ and standard deviation σ of the transfected cells as indicated.
(d) Same as (c), except the plot shows data for TheoAAAAA induced with 1 mM theophylline. Cells in density-contour regions are colored blue-green-yellow in increasing density; number of cells and fraction of cells in the parent gate are as indicated.
(e) Overlay of histograms of mCherry/BFP for the cell populations transfected with TheoAAAAA in 0/1 mM theophylline. One-sample Kolmogorov-Smirnov (KS) test performed in MATLAB with significant α <0.01 confirms the normality assumption of the distribution of log10(mCherry/BFP) for the ribozyme switch, where the KS statistic is 6.7372e-01 for the 0 mM theophylline condition, and is 3.7357e-01 for the 1 mM theophylline condition.

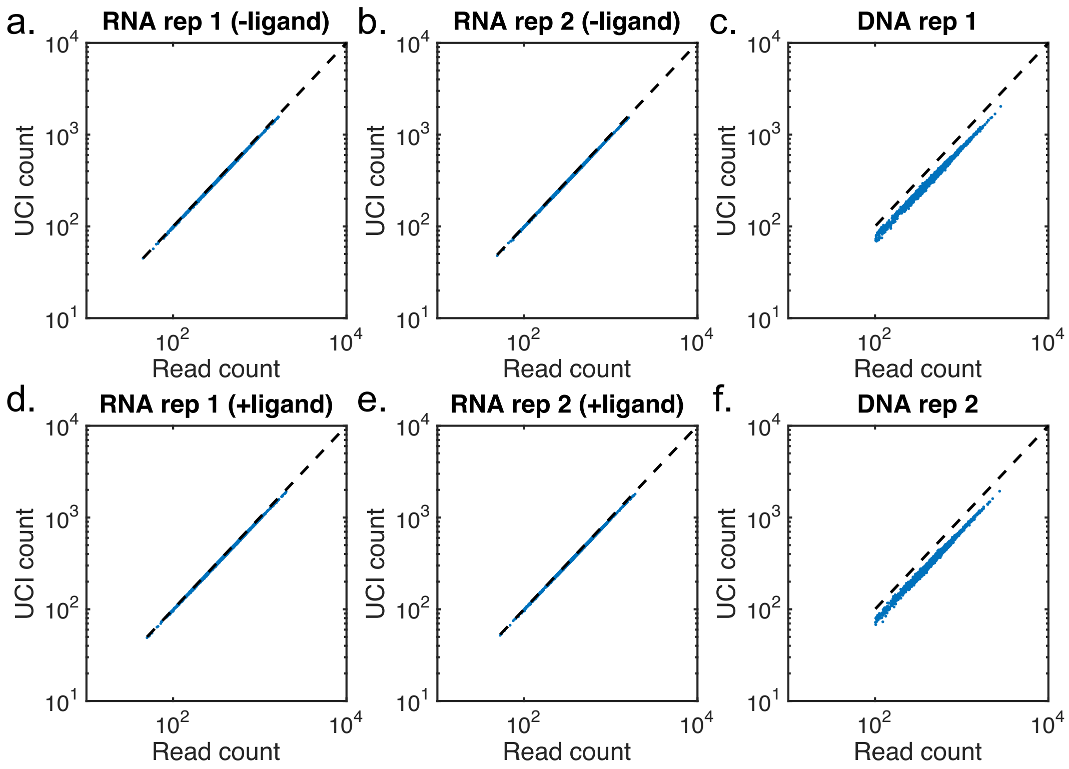

Supplementary Figure 21. Unique Coverage Index (UCI) versus raw sequencing read count of cyclic di-GMP-II ribozyme switch library members from an RNA-Seq experiment. Unique Coverage Index, defined as the unique number of library molecules per sequence. Each dot represents a unique sequence in the library with over 100 read count coverage. Dashed line corresponds to a 1:1 ratio of UCI count versus read count.

(a) UCI count plotted against raw sequencing read count of the recovered RNA library sequence for replicate 1 of the cyclic di-GMP-II ribozyme switch library in 0 mM ligand.

(b) UCI count plotted against raw sequencing read count of the recovered RNA library sequence for replicate 2 of the cyclic di-GMP-II ribozyme switch library in 0 mM ligand.

(c) UCI count plotted against raw sequencing read count of the recovered plasmid DNA library sequence for replicate 1 of the cyclic di-GMP-II ribozyme switch library in 0 mM ligand.

(d) UCI count plotted against raw sequencing read count of the recovered RNA library sequence for replicate 1 of the cyclic di-GMP-II ribozyme switch library in 1 mM ligand.

(e) UCI count plotted against raw sequencing read count of the recovered RNA library sequence for replicate 2 of the cyclic di-GMP-II ribozyme switch library in 1 mM ligand.

(f) UCI count plotted against raw sequencing read count of the recovered plasmid DNA library sequence for replicate 2 of the cyclic di-GMP-II ribozyme switch library in 0 mM ligand.

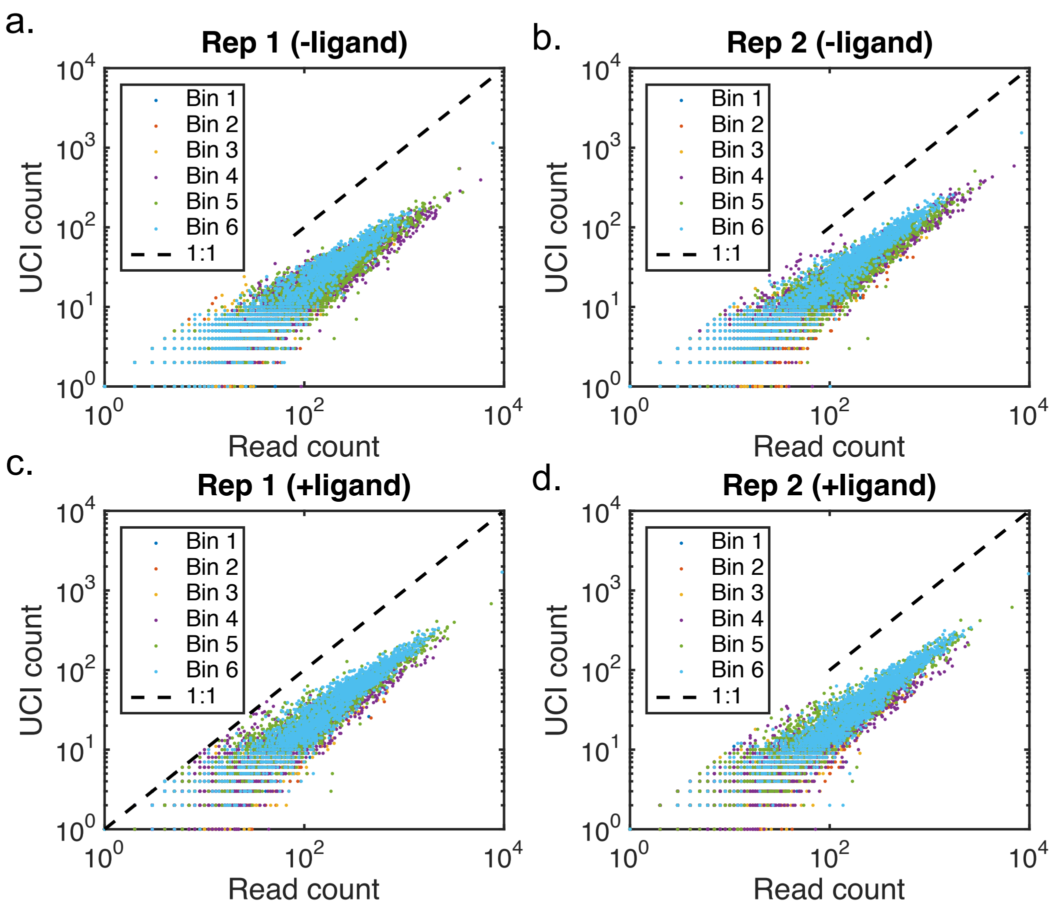

Supplementary Figure 22. Unique Coverage Index (UCI) versus raw sequencing read count of cyclic di-GMP-II ribozyme switch library members from a FACS-Seq experiment. Unique Coverage Index, defined as the unique number of library molecules per sequence. Each dot represents a unique sequence in the library with over 100 read count coverage. Dashed line corresponds to a 1:1 ratio of UCI count versus read count.

(a) UCI count plotted against raw sequencing read count for sequences in each of 6 FACS-sorted bins for replicate 1 of the cyclic di-GMP-II ribozyme switch library in 0 mM ligand.

(b) UCI count plotted against raw sequencing read count for sequences in each of 6 FACS-sorted bins for replicate 2 of the cyclic di-GMP-II ribozyme switch library in 0 mM ligand.

(c) UCI count plotted against raw sequencing read count for sequences in each of 6 FACS-sorted bins for replicate 1 of the cyclic di-GMP-II ribozyme switch library in 1 mM ligand.

(d) UCI count plotted against raw sequencing read count for sequences in each of 6 FACS-sorted bins for replicate 2 of the cyclic di-GMP-II ribozyme switch library in 1 mM ligand.

Supplementary Tables

Supplementary Table 1. List of DNA sequences for library templates and primers.

Name	Description	Sequence (5' → 3')
sTRSVctl_N4	sTRSVctl non-cleaving library	AAACAAACAAA GCTGTCACCGGA TGTGCTT TCCGGTACGTGAGGTCC NNNN GGACGAAACAGC AAAAAGAAAAATAAAAA
Theo_N4-5	Theophylline switch library	AAACAAACAAAGCTGTCACCGGAATACCAGCATCGTCTTGATGCCCTTGGAAG TCCGGTCTGATGAGTCC NNNNN(N) GGACGAAACAGC AAAAAGAAAAATAAAAA
Xan_N5-6	Xanthine switch library	AAACAAACAAAGCTGTCACCGGTGTATTACCCTAGTGGTCG ACCGGTCTGATGAGTCC NNNNN(N) GGACGAAACAGCAAAAAGAAAAATAAAAA
cdiGMPI_N5	cyclic di-GMP-I switch library	AAACAAACAAAGCTGTCACCGCGCACAGGGCAAACCATTCGAAAGAGTGGGACGCAAAGCCTCCGGCCTAAACCAGAAGACATGGTAGGTAGCGGGGTTAC CGCGGTCTGATGAGTCC NNNNN GGACGAAACAGCAAAAAGAAAAATAAAAA
cdiGMPII_N5-6	cyclic di-GMP-II switch library	AAACAAACAAA GCTGTCACCGAGAAACTGTGAAGTATATCTTAAACCTGGGCACTTAAAAGATATATGGAGTTAGTAGTGCAACCTG CTCGGTCTGATGAGTCC NNNNN(N) GGACGAAACAGC AAAAAGAAAAATAAAAA
FA_GTCTN5-6	Folinic acid switch library	AAACAAACAAA GCTGTCACCGGTGCTTGGTACGTTATATTCA GCCGGTCTGATGAGTCT NNNNN(N) AGACGAAACAGC AAAAAGAAAAATAAAAA
pCS4076-N6_ACCG_FWD	Forward primer with N6 UCI (unique coverage index) to PCR library for Gibson assembly into pCS4076	TAACTGATCATAAATATAGGGCCCGGATCC NNNNNN AAACAAACAAAGCTGTCACCG
pCS4076-N6_GTC_REV	Reverse primer with N6 UCI to PCR library for Gibson assembly into	AGGCTGATCAGCGGGTTTGGATCC NNNNNN TTTTTATTTTTCTTTTTGCTGTTTCGTC

	pCS4076	
pCS4077-N4_ACCG_FWD	Forward primer with N4 UCI to PCR library for Gibson assembly into pCS4077	AGCTGTACAAGTAACTGATCATAAGGATCC NNNN AAACAAACAAAGCTGTCACCGG
pCS4077-N4_GTC_REV	Reverse primer with N4 UCI to PCR library for Gibson assembly into pCS4077	AACTAGAAGGCACAGTCGAGGTCTAGA NNNN TTTTTATTTTTCTTTTTGCTGTTTCGTC
pCS408-ACCG_FWD	Forward primer to PCR library for Gibson assembly into pCS408	GCATGGACGAGCTGTACAAGTAACTCGAGAAACAAACAAAGCTGTCACCG
pCS408-GTC_REV	Reverse primer to PCR library for Gibson assembly into pCS408	AGCGGGTTTAAACGGGCCCTCTAGA TTTTTATTTTTCTTTTTGCTGTTTCGTC

N refers to degenerate bases with 25% representation of each of the bases, A, T, C, and G. Parenthesis around N, e.g., NNNN(N), refer to an additional base in the library, that is the library consists of both NNNN and NNNNN.

Supplementary Table 2. List of control ribozymes and individual ribozyme switch sequences.

Name	Description	Sequence (5' → 3')
sTRSV	Control (wild-type) ribozyme	GCUGUCACCGGA UGUGCUU UCCGGUCUGAUGAGUCC GUGA GGACGAAACAGC
sTRSVctl	Control ribozyme	GCUGUCACCGGA UGUGCUU UCCGGUACGUGAGGUCC GUGA GGACGAAACAGC
g814	Control ribozyme	GCUGUCACCGGA UGUU UCCGGUCUGAUGAGUCC AUAA GGACGAAACAGC
g833	Control ribozyme	GCUGUCACCGGA UACU UCCGGUCUGAUGAGUCC CAGA GGACGAAACAGC
g862	Control ribozyme	GCUGUCACCGGA UGCA UCCGGUCUGAUGAGUCC CGCG GGACGAAACAGC
Theo-AAAAA	Theophylline switch	GCUGUCACCGGA AUACCAGCAUCGUCUUGAUGCCCUUGGAAG UCCGGUCUGAUGAGUCC AAAAA GGACGAAACAGC
Theo-CAGAA	Theophylline switch	GCUGUCACCGGA AUACCAGCAUCGUCUUGAUGCCCUUGGAAG UCCGGUCUGAUGAGUCC CAGAA GGACGAAACAGC
Theo-AGAAA	Theophylline switch	GCUGUCACCGGA AUACCAGCAUCGUCUUGAUGCCCUUGGAAG UCCGGUCUGAUGAGUCC AGAAA

		GGACGAAACAGC
Theo-AGGAA	Theophylline switch	GCUGUCACCGGA AUACCAGCAUCGUCUUGAUGCCCUUGGAAG UCCGGUCUGAUGAGUCC AGGAA GGACGAAACAGC
Theo-CAUAA	Theophylline switch	GCUGUCACCGGA AUACCAGCAUCGUCUUGAUGCCCUUGGAAG UCCGGUCUGAUGAGUCC CAUAA GGACGAAACAGC
Theo-CUGAA	Theophylline switch	GCUGUCACCGGA AUACCAGCAUCGUCUUGAUGCCCUUGGAAG UCCGGUCUGAUGAGUCC CUGAA GGACGAAACAGC
Theo-CUUGA	Theophylline switch	GCUGUCACCGGA AUACCAGCAUCGUCUUGAUGCCCUUGGAAG UCCGGUCUGAUGAGUCC CUUGA GGACGAAACAGC
Theo-UGGUA	Theophylline switch	GCUGUCACCGGA AUACCAGCAUCGUCUUGAUGCCCUUGGAAG UCCGGUCUGAUGAGUCC UGGUA GGACGAAACAGC
Theo-CGGCA	Theophylline switch	GCUGUCACCGGA AUACCAGCAUCGUCUUGAUGCCCUUGGAAG UCCGGUCUGAUGAGUCC CGGCA GGACGAAACAGC
Theo-GAAAG	Theophylline switch	GCUGUCACCGGA AUACCAGCAUCGUCUUGAUGCCCUUGGAAG UCCGGUCUGAUGAGUCC GAAAG GGACGAAACAGC
Theo-AUUCA	Theophylline switch	GCUGUCACCGGA AUACCAGCAUCGUCUUGAUGCCCUUGGAAG UCCGGUCUGAUGAGUCC AUUCA GGACGAAACAGC
Theo-AAAGA	Theophylline switch	GCUGUCACCGGA AUACCAGCAUCGUCUUGAUGCCCUUGGAAG UCCGGUCUGAUGAGUCC AAAGA GGACGAAACAGC
Xan-AAGAAG	Xanthine switch	GCUGUCACCGG U GUAUUACCCUAGUGGUCG A CCGGUCUGAUGAGUCU AAGAAG AGACGAAACAGC
Xan-ACGAAG	Xanthine switch	GCUGUCACCGG U GUAUUACCCUAGUGGUCG A CCGGUCUGAUGAGUCU ACGAAG AGACGAAACAGC
Xan-ACGAG	Xanthine switch	GCUGUCACCGG U GUAUUACCCUAGUGGUCG A CCGGUCUGAUGAGUCU ACGAG AGACGAAACAGC
Xan-GAGAAG	Xanthine switch	GCUGUCACCGG U GUAUUACCCUAGUGGUCG A CCGGUCUGAUGAGUCU GAGAAG

		AGACGAAACAGC
Xan-GCGAG	Xanthine switch	GCUGUCACCGG U GUAUUACCCUAGUGGUCG A CCGGUCUGAUGAGUCU GCGAG AGACGAAACAGC
Xan-AAGACG	Xanthine switch	GCUGUCACCGG U GUAUUACCCUAGUGGUCG A CCGGUCUGAUGAGUCU AAGACG AGACGAAACAGC
Xan-GCGUAA	Xanthine switch	GCUGUCACCGG U GUAUUACCCUAGUGGUCG A CCGGUCUGAUGAGUCU GCGUAA AGACGAAACAGC
Xan-GAGUG	Xanthine switch	GCUGUCACCGG U GUAUUACCCUAGUGGUCG A CCGGUCUGAUGAGUCCU GAGUG AGACGAAACAGC
cdGII-CCAAUG	c-di-GMP-II switch	GCUGUCACCG AG AAACUGUGAAGUAUAUCUUAAACCUGGGCACUU AAAAGAUAUAUGGAGUUAGUAGUGCAACCUG CU CGGUCUGAUGAGUCC CCAAUG GGACGAAACAGC
cdGII-CGGAA	c-di-GMP-II switch	GCUGUCACCG AG AAACUGUGAAGUAUAUCUUAAACCUGGGCACUU AAAAGAUAUAUGGAGUUAGUAGUGCAACCUG CU CGGUCUGAUGAGUCC CGGAA GGACGAAACAGC
cdGII-CGUAA	c-di-GMP-II switch	GCUGUCACCG AG AAACUGUGAAGUAUAUCUUAAACCUGGGCACUU AAAAGAUAUAUGGAGUUAGUAGUGCAACCUG CU CGGUCUGAUGAGUCC CGUAA GGACGAAACAGC
cdGII-CGUGAA	c-di-GMP-II switch	GCUGUCACCG AG AAACUGUGAAGUAUAUCUUAAACCUGGGCACUU AAAAGAUAUAUGGAGUUAGUAGUGCAACCUG CU CGGUCUGAUGAGUCC CGUGAA GGACGAAACAGC
Fol-AUAGAG	Folinic acid switch	GCUGUCACCGG U GCUUGGUACGUUAUAUUCA G CCGGUCUGAUGAGUCU AUAGAG AGACGAAACAGC
Fol-UAAGG	Folinic acid switch	GCUGUCACCGG U GCUUGGUACGUUAUAUUCA G CCGGUCUGAUGAGUCU UAAGG AGACGAAACAGC
Fol-UGAGG	Folinic acid switch	GCUGUCACCGG U GCUUGGUACGUUAUAUUCA G CCGGUCUGAUGAGUCU UGAGG AGACGAAACAGC
Fol-UGAUG	Folinic acid switch	GCUGUCACCGG U GCUUGGUACGUUAUAUUCA G CCGGUCUGAUGAGUCU UGAUG AGACGAAACAGC
Fol-UGGAG	Folinic acid switch	GCUGUCACCGG U GCUUGGUACGUUAUAUUCA G CCGGUCUGAUGAGUCU UGGAG AGACGAAACAGC

Fol-UGAAG	Folinic acid switch	GCUGUCACCGG U GCUUGGUACGUUAUAUUCA G CCGGUCUGAUGAGUCU UGAAG AGACGAAACAGC

Supplementary Table 3. RNA-Seq results statistics.

Library	Theophylline	Xanthine	Folinic acid	Folinic acid	Cyclic di-GMP-I	Cyclic di-GMP-II
Loop II	N4-5	N5-6	N5-6	N5-6	N5	N5-6
Gene regulated	mCherry	mCherry	GFP	mCherry	mCherry	mCherry
Ligand conc (mM)	5	5	6	6	1	1
No. sequenced	1280	5120	5120	5120	1024	5120
No. >100 reads	665	5073	5114	5118	1011	4966
R^2 replicates	0.80	0.92	0.89	0.89	0.83	0.85
No. >2 fold AR*	148	1279	510	49	0	113
Max. AR*	6.8	5.6	4.9	2.7	1.9	2.6

* AR, activation ratio

Supplementary Table 4. FACS-Seq results statistics.

Library	Theophylline	Xanthine	Folinic acid	Cyclic di-GMP-II
Loop II	N4-5	N5-6	N5-6	N5-6
Gene regulated	mCherry	mCherry	mCherry	mCherry
Ligand conc (mM)	1	5	6	1
No. sequenced	1280	5117	5102	5100
No. >100 reads	1202	2632	2221	1969
R^2 replicates	0.89	0.93	0.66	0.90
No. >2 fold AR* and p<1e-125	19	203	15	38
Max AR*	3.8	5.0	4.5	2.4

* AR, activation ratio

Supplementary Table 5. List of primers used in reverse transcription and barcoding for multiplexed sequencing.

Name	Sequence (5' → 3')
RT1 (Reverse transcription primer 1)	AGGCTGATCAGCGGGTTTGGTACC NNNNNN TTTTTATTTTTCTTTTTGCTGTTTCGTC
RT2 (Reverse transcription primer 2)	GCGATGCAATTTCCTCATTT
Outer_REV (Outer PCR primer for DNA-seq)	GCGATGCAATTTCCTCATTT
mCherry_fwd (outer PCR primer)	CACCATCGTGGAACAGTACG
EGFP_fwd (outer PCR primer)	CGACAACCACTACCTGAGCA
Illumina i5 forward primer	AATGATACGGCGACCACCGA
Illumina i7 reverse primer	CAAGCAGAAGACGGCATACGAGAT
Barcode primers for pCS4076	
pCS4076-PE1_null_FWD	AATGATACGGCGACCACCGA GATCTACACTCTTTCCCTACACGACGCTCTTCCGAT CT AATATAGGGCCCGGATCC
pCS4076-PE1_C_FWD	AATGATACGGCGACCACCGA GATCTACACTCTTTCCCTACACGACGCTCTTCCGAT CT C AATATAGGGCCCGGATCC
pCS4076-PE1_GC_FWD	AATGATACGGCGACCACCGA GATCTACACTCTTTCCCTACACGACGCTCTTCCGAT CT GC AATATAGGGCCCGGATCC
pCS4076-PE1_ACC_FWD	AATGATACGGCGACCACCGA GATCTACACTCTTTCCCTACACGACGCTCTTCCGAT CT ACC AATATAGGGCCCGGATCC
pCS4076-PE1_TATA_FWD	AATGATACGGCGACCACCGA GATCTACACTCTTTCCCTACACGACGCTCTTCCGAT CT TATA AATATAGGGCCCGGATCC
pCS4076-PE1_GACTC_FWD	AATGATACGGCGACCACCGA GATCTACACTCTTTCCCTACACGACGCTCTTCCGAT CT GACTC AATATAGGGCCCGGATCC
pCS4076-PE1_CGCGAT_FWD	AATGATACGGCGACCACCGA GATCTACACTCTTTCCCTACACGACGCTCTTCCGAT CT CGCGAT AATATAGGGCCCGGATCC
pCS4076-PE1_CAGAAGA_FWD	AATGATACGGCGACCACCGA GATCTACACTCTTTCCCTACACGACGCTCTTCCGAT CT CAGAAGA AATATAGGGCCCGGATCC
pCS4076-PE1_ATGAGACC_FWD	AATGATACGGCGACCACCGA GATCTACACTCTTTCCCTACACGACGCTCTTCCGAT CT ATGAGACC AATATAGGGCCCGGATCC

pCS4076-PE1_TCA_FWD	AATGATACGGCGACCACCGA GATCTACACTCTTTCCCTACACGACGCTCTTCCGAT CT TCA AATATAGGGCCCGGATCC
pCS4076-PE1_CGGC_FWD	AATGATACGGCGACCACCGA GATCTACACTCTTTCCCTACACGACGCTCTTCCGAT CT CGGC AATATAGGGCCCGGATCC
pCS4076-PE1_AAGAG_FWD	AATGATACGGCGACCACCGA GATCTACACTCTTTCCCTACACGACGCTCTTCCGAT CT AAGAG AATATAGGGCCCGGATCC
pCS4076-null_PM2_I1_REV	CAAGCAGAAGACGGCATACGAGAT CGTGAT GTGACTGGAGTTCAGACGTGTGCTCTT CCGATCT TCAGCGGGTTTGGTACC
pCS4076-A_PM2_I2_REV	CAAGCAGAAGACGGCATACGAGAT ACATCG GTGACTGGAGTTCAGACGTGTGCTCTT CCGATCT A TCAGCGGGTTTGGTACC
pCS4076-TT_PM2_I3_REV	CAAGCAGAAGACGGCATACGAGAT GCCTAA GTGACTGGAGTTCAGACGTGTGCTCTT CCGATCT TT TCAGCGGGTTTGGTACC
pCS4076-TCC_PM2_I4_REV	CAAGCAGAAGACGGCATACGAGAT TGGTCA GTGACTGGAGTTCAGACGTGTGCTCTT CCGATCT TCC TCAGCGGGTTTGGTACC
pCS4076-GATA_PM2_I6_REV	CAAGCAGAAGACGGCATACGAGAT ATTGGC GTGACTGGAGTTCAGACGTGTGCTCTT CCGATCT GATA TCAGCGGGTTTGGTACC
pCS4076-TGTGT_PM2_I7_REV	CAAGCAGAAGACGGCATACGAGAT GATCTG GTGACTGGAGTTCAGACGTGTGCTCTT CCGATCT TGTGT TCAGCGGGTTTGGTACC
pCS4076-ATATTG_PM2_I8_REV	CAAGCAGAAGACGGCATACGAGAT ACTTGAGTGACTGGAGTTCAGACGTGTGCTCTTC CGATCT AGCATG TCAGCGGGTTTGGTACC
pCS4076-CGATCCA_PM2_I9_REV	CAAGCAGAAGACGGCATACGAGAT GATCAGGTGACTGGAGTTCAGACGTGTGCTCTTC CGATCT CTGACCA TCAGCGGGTTTGGTACC
pCS4076-GCATGTTT_PM2_I5_REV	CAAGCAGAAGACGGCATACGAGAT ACAGTGGTGACTGGAGTTCAGACGTGTGCTCTTC CGATCT GCATGTTT TCAGCGGGTTTGGTACC
Barcode primers for pCS4077	
pCS4077-PE1_null_FWD	AATGATACGGCGACCACCGAGATCTACACTCTTTCC CTACACGACGCTCTTCCGATCT GTACAAGTAACTGATCATAAGGATCC
pCS4077-PE1_C_FWD	AATGATACGGCGACCACCGAGATCTACACTCTTTCC CTACACGACGCTCTTCCGATCT C GTACAAGTAACTGATCATAAGGATCC
pCS4077-PE1_GC_FWD	AATGATACGGCGACCACCGAGATCTACACTCTTTCC CTACACGACGCTCTTCCGATCT GC GTACAAGTAACTGATCATAAGGATCC

pCS4077-PE1_ACC_FWD	AATGATACGGCGACCACCGAGATCTACACTCTTTCC CTACACGACGCTCTTCCGATCT ACC GTACAAGTAACTGATCATAAGGATCC
pCS4077-PE1_TATA_FWD	AATGATACGGCGACCACCGAGATCTACACTCTTTCC CTACACGACGCTCTTCCGATCT TATA GTACAAGTAACTGATCATAAGGATCC
pCS4077-PE1_GACTC_FWD	AATGATACGGCGACCACCGAGATCTACACTCTTTCC CTACACGACGCTCTTCCGATCT GACTC GTACAAGTAACTGATCATAAGGATCC
pCS4077-PE1_CGCGAT_FWD	AATGATACGGCGACCACCGAGATCTACACTCTTTCC CTACACGACGCTCTTCCGATCT CGCGAT ACAAGTAACTGATCATAAGGATCC
pCS4077-PE1_CAGAAGA_FWD	AATGATACGGCGACCACCGAGATCTACACTCTTTCC CTACACGACGCTCTTCCGATCT CAGAAGA ACAAGTAACTGATCATAAGGATCC
pCS4077-PE1_ATGAGACC_FWD	AATGATACGGCGACCACCGAGATCTACACTCTTTCC CTACACGACGCTCTTCCGATCT ATGAGACC ACAAGTAACTGATCATAAGGATCC
pCS4077-null_PM2_I1_REV	CAAGCAGAAGACGGCATACGAGAT CGTGATGTGACTGGAGTTCAGACGTGTGCTCTTC CGATCT GGCACAGTCGAGGTCTAGA
pCS4077-A_PM2_I2_REV	CAAGCAGAAGACGGCATACGAGAT ACATCGGTGACTGGAGTTCAGACGTGTGCTCTTC CGATCT A GGCACAGTCGAGGTCTAGA
pCS4077-TT_PM2_I3_REV	CAAGCAGAAGACGGCATACGAGAT GCCTAAGTGACTGGAGTTCAGACGTGTGCTCTTC CGATCT TT GGCACAGTCGAGGTCTAGA
pCS4077-TCC_PM2_I4_REV	CAAGCAGAAGACGGCATACGAGAT TGGTCAGTGACTGGAGTTCAGACGTGTGCTCTTC CGATCT TCC GGCACAGTCGAGGTCTAGA
pCS4077-GATA_PM2_I6_REV	CAAGCAGAAGACGGCATACGAGAT ATTGGCGTGACTGGAGTTCAGACGTGTGCTCTTC CGATCT GATA GGCACAGTCGAGGTCTAGA
pCS4077-TGTGT_PM2_I7_REV	CAAGCAGAAGACGGCATACGAGAT GATCTGGTGACTGGAGTTCAGACGTGTGCTCTTC CGATCT TGTGT GGCACAGTCGAGGTCTAGA
pCS4077-ATATTG_PM2_I8_REV	CAAGCAGAAGACGGCATACGAGAT ACTTGAGTGACTGGAGTTCAGACGTGTGCTCTTC CGATCT AGCATG GCACAGTCGAGGTCTAGA
pCS4077-CGATCCA_PM2_I9_REV	CAAGCAGAAGACGGCATACGAGAT GATCAGGTGACTGGAGTTCAGACGTGTGCTCTTC CGATCT CTGACCA GCACAGTCGAGGTCTAGA
pCS4077-GCATGTTT_PM2_I5_REV	CAAGCAGAAGACGGCATACGAGAT ACAGTGGTGACTGGAGTTCAGACGTGTGCTCTTC CGATCT GCATGTTT GCACAGTCGAGGTCTAGA
Barcode primers for pCS408	

pCS408-PE1_null_FWD	AATGATACGGCGACCACCGA GATCTACACTCTTTCCCTACACGACGCTCTTCCGATCT CGAGCTGTACAAGTAACTCGAG
pCS408-PE1_T_FWD	AATGATACGGCGACCACCGA GATCTACACTCTTTCCCTACACGACGCTCTTCCGATCT T CGAGCTGTACAAGTAACTCGAG
pCS408-PE1_GC_FWD	AATGATACGGCGACCACCGA GATCTACACTCTTTCCCTACACGACGCTCTTCCGATCT GC CGAGCTGTACAAGTAACTCGAG
pCS408-PE1_ACC_FWD	AATGATACGGCGACCACCGA GATCTACACTCTTTCCCTACACGACGCTCTTCCGATCT ACC CGAGCTGTACAAGTAACTCGAG
pCS408-PE1_TATA_FWD	AATGATACGGCGACCACCGA GATCTACACTCTTTCCCTACACGACGCTCTTCCGATCT TATA CGAGCTGTACAAGTAACTCGAG
pCS408-PE1_GACTC_FWD	AATGATACGGCGACCACCGA GATCTACACTCTTTCCCTACACGACGCTCTTCCGATCT GACTC CGAGCTGTACAAGTAACTCGAG
pCS408-PE1_TCA_FWD	AATGATACGGCGACCACCGA GATCTACACTCTTTCCCTACACGACGCTCTTCCGATCT TCA CGAGCTGTACAAGTAACTCGAG
pCS408-null_PM2_I1_REV	CAAGCAGAAGACGGCATACGAGAT CGTGAT GTGACTGGAGTTCAGACGTGTGCTCTTCCGATCT GTTTAAACGGGCCCTCTAGA
pCS408-A_PM2_I2_REV	CAAGCAGAAGACGGCATACGAGAT ACATCG GTGACTGGAGTTCAGACGTGTGCTCTTCCGATCT A GTTTAAACGGGCCCTCTAGA
pCS408-TT_PM2_I3_REV	CAAGCAGAAGACGGCATACGAGAT GCCTAA GTGACTGGAGTTCAGACGTGTGCTCTTCCGATCT TT GTTTAAACGGGCCCTCTAGA
pCS408-TCC_PM2_I4_REV	CAAGCAGAAGACGGCATACGAGAT TGGTCA GTGACTGGAGTTCAGACGTGTGCTCTTCCGATCT TCC GTTTAAACGGGCCCTCTAGA
pCS408-GATA_PM2_I6_REV	CAAGCAGAAGACGGCATACGAGAT ATTGGC GTGACTGGAGTTCAGACGTGTGCTCTTCCGATCT GATA GTTTAAACGGGCCCTCTAGA
pCS408-TGTGT_PM2_I7_REV	CAAGCAGAAGACGGCATACGAGAT GATCTG GTGACTGGAGTTCAGACGTGTGCTCTTCCGATCT TGTGT GTTTAAACGGGCCCTCTAGA

Supplementary Table 6. Lentivirus transduction experiment scale for FACS-Seq.

**Library**	**Theophylline**	**Xanthine**	**Xanthine**	**Cyclic di-GMP-II**	**Cyclic di-GMP-II**	**Folinic acid**	**Folinic acid**
**Loop II**	N4-5	N5	N6	N5	N6	N5	N6
**Diversity**	1,280	5,120	4,096	1,024	4,096	1,024	4,096
**Coverage**	400	200	200	200	200	200	200
**Total coverage desired**	512,000	1,024,000	819,200	204,800	819,200	204,800	819,200
**Estimated fraction transduced**	0.1	0.1	0.1	0.1	0.1	0.1	0.1
**Cells to be transduced**	5,120,000	10,240,000	8,192,000	2,048,000	8,192,000	2,048,000	8,192,000
**Cell density per 10 cm dish**	3,480,000	3,480,000	3,480,000	3,480,000	3,480,000	3,480,000	3,480,000
**No. of 10 cm dishes desired**	1.5	2.9	2.4	0.6	2.4	0.6	2.4
**No. of 10 cm dishes used**	2	3	3	1	3	1	3

Supplementary Table 7. FACS-Seq binning sort cell count.

Library	Experiment condition		Number of cells sorted per FACS Gate (P#)						Total cell count sorted
	Rep	Ligand Conc (mM)	P4	P5	P6	P7	P8	P9	
**Theophylline**	1	0	249,946	451,904	257,676	89,239	31,936	7,610	1,088,311
**Theophylline**	2	0	288,793	390,949	259,622	99,416	33,511	5,426	1,077,717
**Theophylline**	1	1	335,452	436,197	202,463	75,533	38,997	7,776	1,096,418
**Theophylline**	2	1	352,298	426,368	192,559	72,889	36,995	7,119	1,088,228
**Xanthine**	1	0	148,143	811,586	1,305,830	833,707	319,809	45,415	3,464,490
**Xanthine**	2	0	175,600	881,457	1,302,051	770,824	284,810	40,822	3,455,564
**Xanthine**	1	5	1,199,702	1,502,041	533,039	252,287	131,738	51,265	3,670,072
**Xanthine**	2	5	1,874,076	1,501,444	548,936	264,138	152,559	67,031	4,408,184
**cyclic di-GMP-II**	1	0	382,763	481,121	312,594	91,994	32,122	8,693	1,309,287
**cyclic di-GMP-II**	2	0	429,470	481,218	288,812	83,868	29,775	7,914	1,321,057
**cyclic di-GMP-II**	1	1	590,158	368,955	152,677	48,145	21,733	6,739	1,188,407
**cyclic di-GMP-II**	2	1	579,887	343,241	142,412	45,584	21,561	6,860	1,139,545
**Folinic acid**	1	0	504,906	816,801	507,149	132,637	32,331	4,829	1,998,653
**Folinic acid**	2	0	581,995	907,121	544,072	140,717	35,277	5,191	2,214,373
**Folinic acid**	1	6	299,412	794,692	767,289	222,165	55,770	7,944	2,147,272
**Folinic acid**	2	6	251,090	703,296	801,100	271,437	63,386	7,740	2,098,049

Supplementary Table 8. List of plasmids used in this work.

Accession	Parent vector	Switch/ribozyme/gene description
pCS4076	pcDNA5/FRT	pcDNA5/FRT_CMV-mCherry-BghA_EF1alpha-BFP
pCS4077	pKL5	pLenti_CMV-mCherry-BghA_EF1alpha-BFP
pCS4078	pCS4077	Control ribozyme sTRSV
pCS4079	pCS4077	Control ribozyme sTRSVctl
pCS4080	pCS4077	Control ribozyme g814
pCS4081	pCS4077	Control ribozyme g833
pCS4082	pCS4077	Control ribozyme g862
pCS4084	pCS4076	Control ribozyme sTRSV
pCS4083	pCS4076	Control ribozyme sTRSVctl
pCS4085	pCS4076	Control ribozyme g814
pCS4086	pCS4076	Control ribozyme g833
pCS4087	pCS4076	Control ribozyme g862
pCS4088	pCS4076	Theophylline switch Theo-AAAAA
pCS4089	pCS4076	Theophylline switch Theo-CAGAA
pCS4090	pCS4076	Theophylline switch Theo-AGAAA
pCS4091	pCS4076	Theophylline switch Theo-AGGAA
pCS4092	pCS4076	Theophylline switch Theo-CAUAA
pCS4093	pCS4076	Theophylline switch Theo-CUGAA
pCS4094	pCS4076	Theophylline switch Theo-CUUGA
pCS4095	pCS4076	Theophylline switch Theo-UGGUA
pCS4096	pCS4076	Theophylline switch Theo-CGGCA

pCS4097	pCS4076	Theophylline switch Theo-GAAAG
pCS4098	pCS4076	Theophylline switch Theo-AUUCA
pCS4099	pCS4076	Theophylline switch Theo-AAAGA
pCS4100	pCS4076	Xanthine switch Xan-AAGAAG
pCS4101	pCS4076	Xanthine switch Xan-ACGAAG
pCS4102	pCS4076	Xanthine switch Xan-ACGAG
pCS4103	pCS4076	Xanthine switch Xan-GAGAAG
pCS4104	pCS4076	Xanthine switch Xan-GCGAG
pCS4105	pCS4076	Xanthine switch Xan-AAGACG
pCS4106	pCS4076	Xanthine switch Xan-GCGUAA
pCS4107	pCS4076	Xanthine switch Xan-GAGUG
pCS4108	pCS4076	c-di-GMP-II switch cdGII-CCAAUG
pCS4109	pCS4076	c-di-GMP-II switch cdGII-CGGAA
pCS4110	pCS4076	c-di-GMP-II switch cdGII-CGUAA
pCS4111	pCS4076	c-di-GMP-II switch cdGII-CGUGAA
pCS4115	pCS4076	Folinic acid switch Fol-AUAGAG
pCS4116	pCS4076	Folinic acid switch Fol-UAAGG
pCS4117	pCS4076	Folinic acid switch Fol-UGAGG
pCS4118	pCS4076	Folinic acid switch Fol-UGAUG
pCS4119	pCS4076	Folinic acid switch Fol-UGGAG
pCS408	pcDNA3.1(+)	EGFP cloned into KpnI/XhoI of pcDNA3.1(+)
pCS4120	pCS408	Control ribozyme sTRSV
pCS4121	pCS408	Control ribozyme sTRSVctl
pCS4122	pCS408	Folinic acid switch Fol-AUAGAG
pCS4123	pCS408	Folinic acid switch Fol-UAAGG
pCS4124	pCS408	Folinic acid switch Fol-UGAGG
pCS4125	pCS408	Folinic acid switch Fol-UGAUG
pCS4126	pCS408	Folinic acid switch Fol-UGGAG
pCS4127	pCS408	Folinic acid switch Fol-UGAAG
pCS4128	pcDNA5/FRT	Human reduced folate carrier 1 SLC19A1
pCS4129	pcDNA5/FRT	Human proton-coupled folate transporter 1 SLC46A1
pCS4130	pcDNA5/FRT	Saccharomyces cerevisiae flr1p
pCS4131	pcDNA5/FRT	Human folate receptor 1 FOLR1
pCS4132	pcDNA5/FRT	Human folate receptor 2 FOLR2
pCS4133	pcDNA5/FRT	Human folate receptor 3 FOLR3
pCS4134	pCDH-EF1α-MCS-(PGK-GFP) Cat #. CD811A-1	Human reduced folate carrier 1 SLC19A1 for lentiviral transduction, copGFP transduction marker
pCS4135	pCDH-EF1α-MCS-(PGK-GFP) Cat #. CD811A-1	Human proton-coupled folate transporter 1 SLC46A1 for lentiviral transduction, copGFP transduction marker

- *E. coli* antibiotic resistance for all plasmids is ampicillin.

Supplementary Table 9. List of folate transporters tested for folinic acid transport. Annotated gene sequence legend: purple, overlap with vector pcDNA5/FRT for Gibson assembly; red, kozak sequence; regular font: coding sequence of the gene; yellow highlight, overlap sequence for assembly of two qBlocks for the coding sequence of the gene; green: 5' UTR or 3'UTR.

Name	Sequence (5' → 3')
Human reduced folate carrier 1 SLC19A1	aaattaatacgactcactatagggagacccaagctggctagcgtttaaacttaagcttggtaccGCCACCatggtgccctccagcccagcggtggagaagcaggtgcccgtggaacctgggcctgaccccgagctccggtcctggcggcacctcgtgtgctacctttgcttctacggcttcatggcgcagatacggccaggggagagcttcatcacccccctacctcctggggcccgacaagaacttcacgcgggagcaggtcacgaacgagatcacgccggtgctgtcgtactcctacctggccgtgctggtgcccgtgttcctgctcaccgactacctgcgctacacgccggtgctgctgctgcaggggctcagcttcgtgtcggtgtggctgctgctgctgctgggccactcggtggcgcacatgcagctcatggagctcttctacagcgtcaccatggccgcgcgcatcgcctattcctcctacatcttctctctcgtgcggcccgcgcgctaccagcgtgtggccggctactcgcgcgctgcggtgctgctgggcgtgttcaccagctccgtgctgggccagctgctggtcactgtgggccgagtctccttctccacgctcaactacatctcgctggccttcctcaccttcagcgtggtcctcgccctcttcctgaagcgccccaagcgcagcctcttcttcaaccgcgacgaccgggggcggtgcgaaacctcggcttcggagctggagcgcatgaatcctggcccaggcgggaagctgggacacgccctgcgggtggcctgtggggactcagtgctggcgcggatgctgcgggagctgggggacagcctgcggcggccgcagctgcgcctgtggtccctctggtgggtcttcaactcggccggctactacctggtggtctactacgtgcacatcctgtggaacgaggtggaccccaccaccaacagtgcgcgggtctacaacggcgcggcagatgctgcctccacgctgctgggcgccatcacgtccttcgccgcgggcttcgtgaagatccgctgggcgcgctggtccaagctgctcatcgcgggcgtcacggccacgcaggcggggctggtcttccttctggcgcacacgcgccacccgagcagcatctggctgtgctatgcggccttcgtgctgttccgcggctcctaccagttcctcgtgcccatcgccacctttcagattgcatcttctctgtctaaagagctctgtgccctggtcttcggggtcaacacgttctttgccaccatcgtcaagaccatcatcactttcattgtctcggacgtgcggggcctgggcctcccggtccgcaagcagttccagttatactccgtgtacttcctgatcctgtccatcatctacttcttgggggccatgctggatggcctgcggcactgccagcggggccaccacccgcggcagcccccggcccagggcctgaggagtgccgcggaggagaaggcagcacaggcactgagcgtgcaggacaagggcctcggaggcctgcagccagcccagagcccgccgctttccccagaagacagcctgggggctgtggggccagcctccctggagcagagacagagcgacccatacctggcccaggccccggccccgcaggcagctgaattcctgagcccagtgacaaccccttcccccctgcactctgtgctccgcccaagcctcaggccctgaggctgcagatgagacttgtccccagctggctgtccatcctcctggtgtcagcaagctgggtttgcagtgtcttccaagcgacggtgttcagaatgtgaaccagtgactctcgggcgcccctgtggtaactttgcaggcggccctcagtgcatccccacgacccctgcctcgagggccgcctgccttagcaatgggggcctccgcttatcctgctagcaggccccctaggattccccctgccctgtgccgcactctggcggtggccacagcgtgctggctcgagtctagagggcccgtttaaacccgctgatcagcct

Human proton-coupled folate transporter 1<br>SLC46A1	agacccaagctggctagcgtttaaacttaagcttggtaccGCCACCatggaggggagcgcgagc<br>cccccggaaaagccccgcgcccgccctgcggctgccgtgctgtgccggggcccggtagagccgct<br>ggtcttcctggccaactttgccttggtcctgcagggcccgctcaccacgcagtatctgtggcaccgcttc<br>agcgccgacctcggctacaatggcacccgccaaaggggggctgcagcaaccgcagcgcggacc<br>ccaccatgcaggaagtggagacccttacctcccactggaccctctacatgaacgtgggcggcttcctg<br>gtggggctcttctcgtccaccctgctgggagcttggagcgacagtgtgggccgccgcccgctgctagt<br>gctggcctcgctgggcctgctgctccaggccctagtgtccgtttttgtggtgcagctgcagctccacgt<br>cggctacttcgtgctgggtcgcatcctttgtgccctcctcggcgacttcggtggccttctggctgctagct<br>ttgcgtccgtggcagatgtcagctccagtcgcagccgcaccttccggatggccctgctggaagccag<br>catcggggtggctgggatgctggcaagcctcctcgggggccactggctccgggcccagggttatgc<br>caaccccttctggctggccttggccttgctgatagccatgactctctatgcagctttctgctttggtgagac<br>cttaaaggagccaaagtccacccggctcttcacgttccgtcaccaccgatccattgtccagctctatgtg<br>gctcccgccccagagaagtccaggaaacatttagccctctactcactggccatcttcgtggtgatcact<br>gtgcactttggggcccaggacatcttaaccctttatgaactaagcacacccctctgctgggactccaaa<br>ctaatcggctatggttctgcagctcagcatctcccctacctcaccagcctgctggccctgaagctcctgc<br>agtactgcctggccgatgcctgggtagctgagatcggcctggccttcaacatcctggggatggtggtc<br>tttgcctttgccactatcacgcctctcatgttcacaggatatgggttgcttttcctgtcattagtcatcacacc<br>tgtcatccgggctaaactctccaagctggtgagagagacagagcagggtgctctcttttctgctgtggc<br>ctgtgtgaatagcctggccatgctgacggcctccggcatcttcaactcactctacccagccactctgaa<br>ctttatgaaggggttccccttcctcctgggagctggcctcctgctcatcccggctgttctgattgggatgc<br>tggaaaaggctgatcctcacctcgagttccagcagtttccccagagcccctgatctgcctggaccaga<br>agacagagggcaagaggagcaaagtgaacaccaagcaactggaggtctgcagctggaagcccag<br>cccctcgagtctagagggcccgtttaaacccgctgatcagcct
Saccharomyces cerevisiae<br>flr1p	agacccaagctggctagcgtttaaacttaagcttggtaccGCCACCatggtctacaccagcaccta<br>cagacacaccatcgtggtggacctgctggaatacctgggcatcgtgtccaacctggaaaccctgcag<br>agcgccagagaggacgagacaagaaagcccgagaacaccgataagaaagagtgcaagcccgact<br>acgacatcgagtgcggccccaatagaagctgcagcgagagcagcaccgacagcgatagctctggc<br>agccagatcgagaagaacgacccccttcagagtggactggaacggccctagcgaccccgagaatcct<br>cagaattggcccctgctgaagaaaagcctggtggtgttccagatcatgctgctgacctgcgtgacctac<br>atgggcagcagcatctacacccctggccaagagtacatccaagaggaattccacgtgggacacgtg<br>gtggccacactgaacctgagcctgtacgtgctcggctatggcctgggacctatcatcttcagccctctg<br>agcgagacagccagatacggcaggctgaacctgtatatggtcaccctgttctttttcatgatcttccaagt<br>gggctgcgccaccgtgcacaatatcggaggactgatcgtgatgcggttcatcagcggcatcctgtgct<br>ctccatctctggcaacaggcggaggaaccgtggccgatatcatcagccctgagatggtgcctctggtg<br>ctcggaatgtggagtgctggtgctgtggctgctcctgttctggctccacttctgggagccgctatggtgg<br>acgccaagaactggcgcttcatcttctggctgctgatgtggctgagcgccgccacctttatcctgctgg<br>cattctttttccccgaaacgcaacaccacaacatcctgtacagacgggccctgaagctgcggaaagag<br>acaggcgacgatcggtactacaccgagcaggacaagctggacagagaagtggatgcccggacctt<br>cctgatcaacaccctgtaccggcctctgaaaatgatcatcaaagagcccgccatcctggccttcgacct<br>gtacattgccgtggcctacggctgcttctacctgttcttcgaggcctttcctatcgtgttcgtgggcatcta<br>ccacttcagtctggtggaagtcggcctggcctatatgggcttctgtgtgggatgtgtgctggcttacggc<br>ctgtttggcatcctgaacatgcggatcatcgtgccccggttcagaaacggcacctttactccagaggca<br>ttcctgatcgtggctatgtgcgtgtgttggtgcctgcctctgtctctgttcctgttcggatggaccgccagg<br>gtgcactggatcctgcctgtgatcagcgaggtgttcttcgtgctggccgtgttcaacatcttccaggcca<br>ccttcgcctacctggccacatgctaccctaaatacgtggcctccgtgttcgccggcaacggcttctgta<br>gagccagcttcgcctgtgcctttccactgttcggcagagctatgtacgacaatctggccaccaagaact<br>accccgtggcctggggatctagcctcgtgggatttctgactctgggcctcgccatcattcccttcatcct<br>gtataagtacggccccagcctgcggaccagaagcagctacacagaggaatgactcgagtctagagg<br>gcccgtttaaacccgctgatcagcct

Human folate receptor 1<br>FOLR1	agacccaagctggctagcgtttaaacttaagcttggtacctggaggcctggctggtgctcacatacaataattaactgctgagtggccttcgcccaatcccaggctccactcctgggctccattcccactccctgcctgtctcctaggccactaaaccacagctgtcccctggaataaggcaagggggagtgtagagcagagcagaagcctgagccagacggagagccacctcctctcccaggaactgaacccaaaggatcacctggtattccctgagagtacagatttctccggcgtggccctcaagggacagacatggctcagcggatgacaacacagctgctgctccttctagtgtgggtggctgtagtaggggaggctcagacaaggattgcatgggccaggactgagcttctcaatgtctgcatgaacgccaagcaccacaaggaaaagccaggccccgaggacaagttgcatgagcagtgtcgaccctggaggaagaatgcctgctgttctaccaacaccagccaggaagcccataaggatgtttcctacctatatagattcaactggaaccactgtggagagatggcacctgcctgcaaacggcatttcatccaggacacctgcctctacgagtgctcccccaacttggggccctggatccagcaggtggatcagagctggcgcaaagagcgggtactgaacgtgcccctgtgcaaagaggactgtgagcaatggtgggaagattgtcgcacctcctacacctgcaagagcaactggcacaagggctggaactggacttcagggttaacaagtgcgcagtgggagctgcctgccaacctttccatttctacttccccacacccactgttctgtgcaatgaaatctggactcactcctacaaggtcagcaactacagccgagggagtggccgctgcatccagatgtggttcgacccagcccagggcaaccccaatgaggaggtggcgaggttctatgctgcagccatgagtggggctgggccctgggcagcctggcctttcctgcttagcctggccctaatgctgctgtggctgctcagctgacctccttttaccttctgatacctggaaatccctgccctgttcagccccacagctcccaactatttggttcctgctccatggtcgggcctctgacagccactttgaataaaccagacaccgcacatgtgtcttgagaattatttggaaaaaaaaaaaaaaaaaaactcgagtctagagggcccgtttaaacccgctgatcagcct
Human folate receptor 2<br>FOLR2	agacccaagctggctagcgtttaaacttaagcttggtaccatttcactcagtgcttaccagagcgcgttgtctaccctgtaccgaagacagaggctgtggggacagcctaggggcctggatctattgcctacttagagagaggccaactcagacacagccgtgtatgctcccagcagcaacggaggttcagctccgcctgcagggacagaaagacatggtctggaaatggatgccacttctgctgcttctggtctgtgtagccaccatgtgcagtgcccaggacaggactgatctcctcaatgtctgtatggatgccaagcaccacaagacaaagccaggtcctgaggacaagctgcatgaccaatgcagtccctggaagaagaatgcctgctgcacagccagcaccagccaggagctgcacaaggacacctcccgcctgtacaactttaactgggaccactgcggcaagatggagcccgcctgcaagcgccacttcatccaggacacctgtctctatgagtgctcacccaacctggggccctggatccagcaggtgaatcagagctggcgcaaagaacgcttcctggatgtgcccttatgcaaagaggactgtcagcgctggtgggaggattgtcacacctcccacacgtgcaagagcaactggcacagaggatgggactggacctcaggagttaacaagtgcccagctggggctctctgccgcacctttgagtcctacttccccactccagctgccctttgtgaaggcctctggagtcactcatacaaggtcagcaactacagccgagggagcggccgctgcatccagatgtggtttgattcagcccagggcaaccccaacgaggaagtggcgaggttctatgctgcagccatgcatgtgaatgctggtgagatgcttcatgggactgggggtctcctgctcagtctggccctgatgctgcaactctggctccttggctgagttcagtcctcccagactacctgccctcagcttggataaccaggctgggctcagctcagctcccacaaatgacagccccttaagcatgcttctattagtcacctaaccctctgtcacccagtctgttgctgctccatggtggggccaagagtcacttctaataaacagactgttttctaataattccaaaaaaaaaaaaaaaaactcgagtctagagggcccgtttaaacccgctgatcagcct
Human folate receptor 3<br>FOLR3	agacccaagctggctagcgtttaaacttaagcttggtaccgagcaagctggtgtcagagcctggacctacagcgctgttggtggaggtcctgcctccaggaatagatggacatggcctggcagatgatgcagctgctgcttctggctttggtgactgctgcggggagtgcccagcccaggagtgcgcgggccaggacggacctgctcaatgtctgcatgaacgccaagcaccacaagacacagcccagccccgaggacgagctgtatggccagtgcagtccctggaagaagaatgcctgctgcacggccagcaccagccaggagctgcacaaggacacctcccgcctgtacaactttaactgggatcactgtggtaagatggaacccacctgcaagcgccactttatccaggacagctgtctctatgagtgctcacccaacctggggccctggatccggcaggtcaaccagagctggcgcaaagagcgcattctgaacgtgcccctgtgcaaagaggactgtgagcgctggtgggaggactgtcgcacctcctacacctgcaaaagcaactggcacaaaggctggaattggacctcagggattaatgagtgtccggccggggccctctgcagcacctttgagtcctacttccccactccagccgccctttgtgaaggcctctggagccactccttcaaggtcagcaactatagtcgagggagcggccgctgcatccagatgtggtttgactcagcccagggcaaccccaatgaggaggtggccaagttctatgctgcggccatgaatgctggggccccgtctcgtgggattattgattcctgatccaagaagggtcctctggggttcttccaacaacctattctaatagacaaatccacatgtgaaaaaaaaaaaaaaaaaaactcgagtctagagggcccgtttaaacccgctgatcagcct

Supplementary Note 1. FACS-Seq noise

Sequencing read depth is typically correlated with data quality. The FACS-Seq and RNA-Seq assays had the same sequencing coverage; however, the data quality was poorer for FACS-Seq than RNA-Seq (Figure 2e, Figure 3c). For both FACS-Seq and RNA-Seq, sequences were filtered with a cutoff of 100 reads under each ligand and replicate condition, which was empirically determined to result in good replicate agreement for RNA-Seq (Supplementary Figure 3). In addition, the median read count coverage of the same libraries was slightly higher in FACS-Seq than in RNA-Seq. Thus, the assays should exhibit similar measurement accuracy, if the nature of the source of dispersion is similarly random. A directly comparable FACS-Seq and RNA-Seq experiment with flanking unique coverage index (UCI, consisting of 4 to 6 random bases on each side of the switch insert, and added on during the library construction step (Supplementary Figure 1a)) was performed to monitor for loss in library sequence coverage and diversity from the start of the library construction process through to the end of the sequencing results. For RNA-Seq, the UCI count was nearly identical to the read count for both RNA and DNA sequences (Supplementary Figure 21). In contrast, the UCI count for FACS-Seq sequences was 10-fold less than the sequence read count (Supplementary Figure 22), indicating a substantial loss in sampling coverage of library members in the library propagation, FACS, and sequencing process.

The FACS-Seq assay exhibits greater noise in the resulting measurements compared with the RNA-Seq assay likely due to the former's reliance on lentiviral library transduction. Lentiviral transduction allows for the propagation of library DNA over many cell generations, and ensures one integration event per cell via a Poisson process for uniquely linking genotype to phenotype[1]. However, recent reports indicate that lentiviral transduction exhibits a high rate of recombination events due to the pseudo-double stranded lentiviral particle genome, resulting in

higher rates of template switching[2,3]. The recombination events can result in silenced expression of one of the fluorescent proteins unrelated to the activity of the encoded ribozyme switch. The workflow also requires enrichment for lower basal expression constructs by an initial sorting round which can inadvertently enrich for constructs in which reporter expression is silenced by mechanisms other than ribozyme self-cleavage. Another source of noise can be associated with the much longer duration of the FACS-Seq experiment versus the RNA-Seq experiment (~30 days versus ~3 days for template DNA to be in a cellular context), from lentiviral production to multiple rounds of sorting and cell passaging, resulting in more time for mutations to accumulate in the cell lines outside the sequenced region.

Supplementary Note 2. Data de-noising using AutoML

We compared RNA-Seq results to FACS-Seq results in order to determine if RNA-Seq results are predictive of FACS-Seq results for the sequence-dependent regulatory activity of ribozyme switches. However, the higher noise levels from the FACS-Seq results prevented any conclusions from being drawn with confidence. Data noise reduction is possible if there is sufficient information in the FACS-Seq results for some underlying sequence-function relationships to be learned, such that the subset of data with higher quality can reinforce a model that learned these principles, while excluding data that are random noise. We used an automated implementation of several regression models using the AutoML algorithm in an R implementation of H2O.ai, including generalized linear model, distributed random forests, gradient boosting machine (tree-based ensemble model), and deep learning. If sequences within a switch library share an overarching set of principles that determine its sequence-dependent gene-regulatory activity, a model that learns this set of principles will be more robust to experimental noise, i.e., effectively "denoising" the data. To prepare the data, sequence identity and position are transformed into one-hot encoded 1's and 0's at each base and position, and normalized RNA levels and relative fluorescence levels in the no-ligand condition, and activation ratios are standardized such that the mean is rescaled to zero and the standard deviation is rescaled to 1. In each of theophylline, xanthine, folinic acid, and cyclic di-GMP libraries, denoising with AutoML significantly increased the fraction of sequences identified by both RNA-Seq and FACS-Seq as having high activation ratios (Supplementary Figure 6).

To further investigate the learned sequence motifs by the model, we performed gradient boosting tree regression (GBM) for xanthine and theophylline libraries on RNA-Seq normalized RNA levels and FACS-Seq relative fluorescence values. The gradient boosting machine (GBM) and deep learning consistently outperform the other models in AutoML. While deep learning models typically perform a few percentage points better than GBM, training deep learning

models requires significantly greater computing time and power. GBM can quickly extract sequence-function relationships without compromising performance. For both theophylline and xanthine libraries, we found that GBM effectively improves the replicate correlation of RNA-Seq and FACS-Seq data (Supplementary Figure 5a-c, f-h). This results in a greater number of sequences being characterized with greater quantitative accuracy. Without denoising, only 665 theophylline sequences with >100 read count coverage were characterized with RNA-Seq for a replicate $R^2 = 0.80$. After GBM denoising, 1,275 unique sequences with just >20 reads show a replicate correlation of $R^2 = 0.91$ (Supplementary Figure 5a). The improvement in FACS-Seq correlation is less pronounced (Supplementary Figure 5b), but the model drastically improves the correlation between RNA-Seq and FACS-Seq results, raising R^2 from 0.34 to 0.74 (Supplementary Figure 5c). It can be seen from the plot comparing FACS-Seq and RNA-Seq that this improvement in correlation is a result of identifying and relabeling the subset of sequences that have low mCherry/BFP ratios but high RNA/DNA ratios. The model has likely learned some underlying sequence-function principles that predict these sequences to give high mCherry/BFP ratios, as the RNA-Seq results would suggest. The improvement on replicate correlation by denoising also lends support to the hypothesis that the greater noise in the FACS-Seq data could be a result of silenced mCherry expression in the lentiviral library-transduced cells. Even though the model does not correlate as strongly with the original raw data (Supplementary Figure 5d), the fact that replicate measurements from independently trained models demonstrate stronger correlation is evidence for the model picking up on underlying motifs (Supplementary Figure 5e). For the xanthine library, replicates for both RNA-Seq and FACS-Seq are strongly correlated due to greater sequencing depth and coverage, which does not leave much room for the denoising improvement. However, as with the theophylline library, there is a sharp increase in correlation between RNA-Seq and FACS-Seq, along with the removal of the "silenced" sequences (Supplementary Figure 5f-j).

GBM models are complex ensembles of weaker models, and are consequently not very interpretable beyond the relative importance metric of each position and base in contributing to function. The relative importance pattern of the sequence-position features agree strongly between replicates and remain similar between RNA-Seq and FACS-Seq experiments, which can account for, at least in part, the improved correlation between the two assay results (Supplementary Figure 5e). Other than the position in the last base of loop II, the relative importance profiles do not agree completely with the entropy analyses of the top 5th percentile of sequences with the lowest mRNA and protein expression levels. Future studies to improve the interpretability of GBMs could improve our understanding on how the sequence features identified herein with greater relative importance contribute to sequence-dependent ribozyme switches function.

Supplementary Note 3. Folinic acid ribozyme switches interact with the mCherry coding sequence leading to misfolding

Engineered molecular devices can interact with surrounding sequences in undesired ways, and care needs to be taken to ensure the modularity of the engineered device[4,5]. When the folinic acid switch library was first screened in RNA-seq along with the other switch libraries in the mCherry vector, the library activity exhibited higher basal expression than the other libraries (Supplementary Figure 12a), even though bulk library *in vitro* cleavage, which was performed without the mCherry sequence, was comparable to the other switch libraries (Supplementary Figure 8). The activation ratio for individually assayed mCherry-linked folinic acid switches reached up to 1.9 fold (Supplementary Figure 12b); however, the same switches when used to control eGFP-expression exhibit activation ratios up to 5.3 fold (Figure 4k). Computational folding of the mCherry-linked ribozyme switch suggested that part of the folinic acid aptamer and sTRSV ribozyme stem III can interact with the mCherry coding sequence (Supplementary Figure 12c). The RNA-Seq assay was repeated for the folinic acid library inserted in the 3' UTR of an enhanced green fluorescent protein (eGFP) reporter, which is not predicted to similarly misfold with the folinic acid switch sequence. Our data show that basal RNA expression of the eGFP-linked folinic acid switch was decreased for every library member (Supplementary Figure 12d) by up to ~2.8 fold. The decrease in basal RNA expression leads to increased activation ratios of normalized RNA levels as measured by RNA-Seq by up to ~2 fold (Supplementary Figure 12e).

References

1. Fehse, B., Kustikova, O. S., Bubenheim, M. & Baum, C. Pois(s)on – It's a Question of Dose…. *Gene Ther.* **11**, 879–881 (2004).
2. Inoue, F. *et al.* A systematic comparison reveals substantial differences in chromosomal versus episomal encoding of enhancer activity. *Genome Res.* **27**, 38–52 (2017).
3. Murtha, M. *et al.* FIREWACh: high-throughput functional detection of transcriptional regulatory modules in mammalian cells. *Nat. Methods* **11**, 559–565 (2014).
4. Kosuri, S. *et al.* Composability of regulatory sequences controlling transcription and translation inEscherichia coli. *Proceedings of the National Academy of Sciences* **110**, 14024–14029 (2013).
5. Lou, C., Stanton, B., Chen, Y.-J., Munsky, B. & Voigt, C. A. Ribozyme-based insulator parts buffer synthetic circuits from genetic context. *Nat. Biotechnol.* **30**, 1137–1142 (2012).
