# Supplementary material for: Massively parallel RNA device engineering in mammalian cells with RNA-Seq
Source: Nat Commun. 2019 Sep 23;10:4327. doi: 10.1038/s41467-019-12334-y (PMC6757056; doi:10.1038/s41467-019-12334-y)
Supplement: Supplementary file 3 — Reporting Summary [file 41467_2019_12334_MOESM3_ESM.pdf]

nature research

Corresponding author(s): Christina D. Smolke

Reporting Summary

Nature Research wishes to improve the reproducibility of the work that we publish. This form provides structure for consistency and transparency in reporting. For further information on Nature Research policies, see Authors & Referees and the Editorial Policy Checklist.

Statistical parameters

When statistical analyses are reported, confirm that the following items are present in the relevant location (e.g. figure legend, table legend, main text, or Methods section).

n/a	Confirmed	
[ ]	[x]	The exact sample size (*n*) for each experimental group/condition, given as a discrete number and unit of measurement
[ ]	[x]	An indication of whether measurements were taken from distinct samples or whether the same sample was measured repeatedly
[ ]	[x]	The statistical test(s) used AND whether they are one- or two-sided<br>*Only common tests should be described solely by name; describe more complex techniques in the Methods section.*
[x]	[ ]	A description of all covariates tested
[ ]	[x]	A description of any assumptions or corrections, such as tests of normality and adjustment for multiple comparisons
[ ]	[x]	A full description of the statistics including central tendency (e.g. means) or other basic estimates (e.g. regression coefficient) AND variation (e.g. standard deviation) or associated estimates of uncertainty (e.g. confidence intervals)
[ ]	[x]	For null hypothesis testing, the test statistic (e.g. *F*, *t*, *r*) with confidence intervals, effect sizes, degrees of freedom and *P* value noted<br>*Give P values as exact values whenever suitable.*
[x]	[ ]	For Bayesian analysis, information on the choice of priors and Markov chain Monte Carlo settings
[x]	[ ]	For hierarchical and complex designs, identification of the appropriate level for tests and full reporting of outcomes
[ ]	[x]	Estimates of effect sizes (e.g. Cohen's *d*, Pearson's *r*), indicating how they were calculated
[ ]	[x]	Clearly defined error bars<br>*State explicitly what error bars represent (e.g. SD, SE, CI)*

Our web collection on statistics for biologists may be useful.

Software and code

Policy information about availability of computer code

Data collection	Custom code was used to generate gates used in the 6-way binning sorts of FACS-Seq experiments, and the scripts are found at https://github.com/jsxiang/Influx-custom-gates. All other data collection uses the instruments' commercial software.
Data analysis	Custom scripts used for processing Illumina sequencing data are found at https://github.com/jsxiang/processFASTQ. Custom scripts used in analyzing RNA-Seq results are found at https://github.com/jsxiang/Ribozyme-RNA-seq. Custom scripts used in analyzing FACS-Seq results are found at https://github.com/jsxiang/Ribozyme-FACS-seq. Custom code used in bioinformatics analyses are found at https://github.com/jsxiang/Ribozyme-SeqFun.

For manuscripts utilizing custom algorithms or software that are central to the research but not yet described in published literature, software must be made available to editors/reviewers upon request. We strongly encourage code deposition in a community repository (e.g. GitHub). See the Nature Research guidelines for submitting code & software for further information.

Data

Policy information about availability of data

All manuscripts must include a data availability statement. This statement should provide the following information, where applicable:

- Accession codes, unique identifiers, or web links for publicly available datasets
- A list of figures that have associated raw data
- A description of any restrictions on data availability

The authors declare that the RNA-Seq, FACS-Seq and flow cytometry data supporting the findings of this study are available in the following supplementary information files:

SourceData1_Figure2and3_Theophylline_seq.csv
SourceData2_Figure2and3_Theophylline_validation.csv
SourceData3_Figure3e_Theophylline_min_20reads_seq.csv
SourceData4_Figure4d,g_Xanthine_seq.csv
SourceData5_Figure4j_Xanthine_validation.csv
SourceData6_Figure4e_folinic-acid_eGFP_seq.csv
SourceData7_Figure4h_folinic-acid_mCherry_seq.csv
SourceData8_Figure4k_folinic-acid_validation.csv
SourceData9_Figure4f,i_cdGII_seq.csv
SourceData10_Figure4l_cdGII_validation.csv
SourceData11_SuppFigure9d,12a,b_cdGI_seq.csv
SourceData12_Figure2e_TheoDoseResponse.csv

Sequencing data are found on the NCBI Sequence Read Archive with accession numbers SAMN12605057 for Theophylline Switch RNAseq, SAMN12605058 for Folinic Acid and Xanthine RNAseq, SAMN12605059 for Cyclic di-GMP RNAseq, SAMN12605060 for Theophylline and cyclic di-GMP FACSseq, SAMN12605061 for Xanthine FACSseq and SAMN12605062 for Folinic Acid FACSseq.

The following plasmids are deposited on Addgene: pCS4077 (131738), pCS4083 (131739), pCS4084 (131740), pCS4092 (131741), pCS4102 (131742), pCS4110 (131743), pCS4120 (131744), pCS4121 (131745), pCS4127 (131746), pCS4134 (131747).

All other data are available from the authors upon request.

Field-specific reporting

Please select the best fit for your research. If you are not sure, read the appropriate sections before making your selection.

[x] Life sciences   [ ] Behavioural & social sciences   [ ] Ecological, evolutionary & environmental sciences

For a reference copy of the document with all sections, see nature.com/authors/policies/ReportingSummary-flat.pdf

Life sciences study design

All studies must disclose on these points even when the disclosure is negative.

Sample size	For RNA-Seq experiments, two biological replicates from separate transfection experiments are used. For FACS-Seq experiments, two replicates from separate 6-way binning sorting experiments are used. Considering the high cost of sequencing and FACS sorting, more replicates are not used; strength of replicate correlation is reported in the main and supplementary figures to show R^2 >0.80. For qPCR experiments, three technical replicates from separate qPCR wells are used. For flow cytometry experiments, two to four replicates from separate transfection experiments are used. Due to the large number of individual samples tested in multiple ligand conditions, more replicates are not included. There is also a high level of agreement between replicates given the tight error bars, as shown in all flow cytometry results.
Data exclusions	No data was excluded.
Replication	Replicates of RNA-Seq experiments are separate plasmid transfection and flow cytometry analysis performed in parallel. Replicates of FACS-Seq experiments are the same transformed library sorted into 6-way bins in parallel. Replicates of qPCR experiments are repeat measurements of the same reverse transcribed cDNA. Replicates of flow cytometry experiments are separate plasmid transfection and flow cytometry performed in parallel.
Randomization	All samples are allocated randomly.
Blinding	Blinding of ligand conditions used in switch induction was not performed in any of the experimental analysis because negative controls are included. Blinding is effectively in place for sequencing experiments.

Reporting for specific materials, systems and methods

Materials & experimental systems

n/a	Involved in the study	
[ ]	[x]	Unique biological materials
[x]	[ ]	Antibodies
[ ]	[x]	Eukaryotic cell lines
[x]	[ ]	Palaeontology
[x]	[ ]	Animals and other organisms
[x]	[ ]	Human research participants

Methods

n/a	Involved in the study	
[x]	[ ]	ChIP-seq
[ ]	[x]	Flow cytometry
[x]	[ ]	MRI-based neuroimaging

Unique biological materials

Policy information about availability of materials

Obtaining unique materials	All unique materials used are readily available from the authors.

Eukaryotic cell lines

Policy information about cell lines

Cell line source(s)	HEK293T cells from ATCC have been used in all cell culture experiments.
Authentication	Cell lines used were not authenticated.
Mycoplasma contamination	Cell lines were not tested for mycoplasma contamination.
Commonly misidentified lines<br>(See ICLAC register)	HEK293T cells are not found on the ICLAC register as a commonly misidentified cell line.

Flow Cytometry

Plots

Confirm that:

[x] The axis labels state the marker and fluorochrome used (e.g. CD4-FITC).

[x] The axis scales are clearly visible. Include numbers along axes only for bottom left plot of group (a 'group' is an analysis of identical markers).

[x] All plots are contour plots with outliers or pseudocolor plots.

[x] A numerical value for number of cells or percentage (with statistics) is provided.

Methodology

Sample preparation	Cells from culture are trypsinized, rinsed with 1XPBS, before being resuspended with growth media DMEM+10 % FBS and 1% pen/strep. Cells are handled at room temperature.
Instrument	The analyzer used in all experiments is the MACSQuant VYB from Miltenyi Biotec. The sorter used is the BD Influx cell sorter for 6-way binning sorts or the BD FACSAria II for single bin sorts.
Software	Custom code is used to analyze flow cytometry data and can be found at https://github.com/jsxiang/FlowAnalysis.
Cell population abundance	The number of cells sorted from the 6-way sorts are indicated in Supplementary Table 7.
Gating strategy	Analysis of flow cytometry data was performed using a custom MATLAB script, available at https://github.com/jsxiang/FlowAnalysis, where cells are gated for viable (3.5<log10(FSC-A)<4.6, and 4.6< log10(SSC-A)<5.05) and singlets (log10(FSC-H)<0.65*log10(FSC-A)), and transfected cells (BFP > 10^2.7 fluorescence units are used in downstream analysis), see Supplementary Figure 19.

[x] Tick this box to confirm that a figure exemplifying the gating strategy is provided in the Supplementary Information.
